# Herb-derived immunometabolic modulators: traditional Chinese medicine at the crossroads of metabolism and antitumor immunity

**DOI:** 10.1186/s13020-026-01448-3

**Published:** 2026-06-26

**Authors:** Bo Zhang, Na Wang, Xiaoshan Wang, Xuerui Wang, Lihan Shang, Bingsheng Sun, Fanming Kong

**Affiliations:** 1 Department of Oncology, First Teaching Hospital of Tianjin University of Traditional Chinese Medicine, Tianjin, 300381 China; 2 National Clinical Research Center for Chinese Medicine, Tianjin, 300381 China; 3 Tianjin Cancer Institute of Traditional Chinese Medicine, Tianjin, 300381 China; 4 Department of Lung Cancer, Tianjin Medical University Cancer Institute and Hospital, National Clinical Research Center for Cancer, Tianjin, 300060 China; 5 Tianjin Clinical Research Center for Cancer and Key Laboratory of Cancer Prevention and Therapy, Tianjin, 300060 China

**Keywords:** Traditional Chinese medicine, Immunometabolism, Tumor microenvironment, Glycolysis and lactate, Lipid and bile-acid metabolism, Ferroptosis and immunogenic cell death, Dendritic cells, Tumor-associated macrophages, Myeloid-derived suppressor cells

## Abstract

**Background:**

Traditional Chinese Medicine (TCM) has long been applied in oncology to “support vital Qi and eliminate pathogenic factors”, yet its place within modern immunometabolic therapy is still not clearly defined. Tumor, stromal, and immune cells are now understood to be organized around several recurrent metabolic axes, including glycolysis–lactate, mitochondrial stress and immunogenic cell death (ICD), lipid–bile-acid signaling, and redox balance. Clarifying how herb-based formulas, isolated compounds, and contemporary delivery systems influence these axes may provide a mechanistic foundation for integrating TCM into precision cancer treatment.

**Main body:**

This narrative review brings together ethnopharmacological knowledge with pharmacological and mechanistic studies, omics-based profiling, and emerging nanomedicine reports that examined TCM-derived interventions with defined metabolic and immune outcomes in solid tumors. We first outline how metabolic reprogramming of the tumor microenvironment (TME) shapes major immune populations, including dendritic cells (DCs), CD8⁺ T cells, tumor-associated macrophages (TAMs), myeloid-derived suppressor cells (MDSCs), and NK/NKT cells. We then arrange representative herb-derived agents along four immunometabolic axes. Across Axis I–IV, multiple prescriptions and monomers have been reported to attenuate tumor glycolytic flux and lactate burden, induce mitochondrial damage and ferroptosis-linked ICD, normalize lipid–bile-acid-centered myeloid niches, and improve DC and T-cell metabolic fitness. Examples include Astragalus-based formulas, Gegen Qinlian decoction (GQD), ginsenosides, berberine, licochalcone A, emodin, celastrol-Rg3 and iron-based nanoplatforms, Jianpi Jiedu and Jianpi Huayu decoctions, Compound Kushen Injection, Compound Fuling Granule, Hochu-ekki-to, Kejinyan decoction, Huaier, Ganoderma polysaccharides, Shenqi Yiqi Capsule, and polysaccharide-loaded vesicles or microneedles. Finally, we relate these axes to classical TCM doctrines such as *Fuzheng Quxie*, *Tiaogan Hepi*, and *Peiben Chuzhuo*, proposing a clinically oriented, syndrome-informed framework.

**Conclusion:**

TCM-derived interventions can be systematically positioned along four convergent immunometabolic axes that coordinate interactions between tumors and the immune system. Considering TCM as an immunometabolic co-therapy highlights its potential to convert “cold” tumors into “hot” lesions, deepen responses to chemo-, radio- and immunotherapy, and, in some contexts, improve treatment tolerance. Future studies should emphasize rigorous mechanistic dissection, standardized and chemically defined formulations, biomarker-guided patient selection, and well-designed prospective clinical trials to translate this axis-based framework into precision integrative oncology. However, most TCM-derived immunometabolic interventions remain incompletely validated, and their translation will require axis-matched biomarkers, rigorous safety assessment, and prospective clinical validation.

## Highlights

- TCM-based interventions can be organized along four major immunometabolic axes.
- Axis I–IV connect glycolysis and lactate, ICD/ferroptosis, lipid–bile-acid signaling, and DC/T-cell metabolic fitness.
- Herbal formulas and monomers remodel tumor–immune metabolic crosstalk within the tumor microenvironment.
- Classical TCM doctrines can be reinterpreted through modern immunometabolic concepts.
- Key gaps in mechanistic understanding and clinical translation of TCM-derived immunometabolic strategies are discussed.

## Introduction

Solid tumors evolve within a metabolically dynamic milieu in which cancer cells both compete with and actively reprogram neighboring immune and stromal populations [1, 2]. Persistent aerobic glycolysis, mitochondrial dysfunction, distortions in lipid and bile-acid pathways, and chronic redox stress together reshape the bioenergetic “rules” that govern dendritic cells (DCs), T cells, and myeloid subsets, ultimately favoring immune evasion and treatment resistance [3–6]. Across multiple malignancies, a glycolysis-dominant, lactate-enriched state is associated with poor immune-cell infiltration and adverse outcomes, even when tumor antigens and pharmacological checkpoint targets are abundantly expressed [7–10].

A further layer of immunosuppression is imposed by dysregulated lipid, bile-acid, and amino-acid metabolism. Enhanced lipid uptake and storage in tumor-associated macrophages (TAMs) and myeloid-derived suppressor cells (MDSCs) stabilize anti-inflammatory programs and dampen antigen presentation, while bile-acid and tryptophan–aryl hydrocarbon receptor (AhR) signaling along the gut–liver axis reinforce tolerance, particularly in hepatocellular carcinoma and colorectal cancer [3, 11–13]. In parallel, mitochondrial integrity, susceptibility to ferroptosis, and local redox balance determine whether dying tumor cells emit sufficient “danger signals” to elicit immunogenic cell death (ICD) and DC cross-priming, or are silently cleared with little immune activation [14–18].

Attempts to manipulate these processes using synthetic inhibitors directed at individual metabolic nodes—such as specific glycolytic enzymes, lipid-synthesis pathways, or bile-acid receptors—have yielded important mechanistic insights but generally modest clinical benefit when used as monotherapies [11, 18]. Tumors frequently adapt by exploiting pathway redundancy, metabolic plasticity, and spatial heterogeneity, and systemic inhibition of central metabolic routes can simultaneously compromise proliferating immune cells. These limitations have intensified interest in multi-target agents and delivery platforms designed to influence several convergent axes at once, ideally with some degree of spatial or cell-type selectivity [19–22].

Traditional Chinese Medicine (TCM) occupies a distinctive position in this context. Classical formulas were assembled empirically to correct systemic “patterns” such as “spleen deficiency,” “liver constraint,” or “damp-heat,” which can now be reinterpreted as constellations of metabolic and inflammatory abnormalities [1, 23–25]. Network pharmacology, omics analyses, and experimental studies indicate that several prescriptions can simultaneously modulate glycolysis, mitochondrial activity, lipid and bile-acid circuits, and redox balance, thereby reshaping both tumor and immune compartments [26–28]. Systems-biology work on Kejinyan decoction, Xihuang Pill, and related formulas, for example, has revealed coordinated effects on the tumor microenvironment (TME), angiogenesis, and immune regulation [21, 29–31].

In parallel, a new generation of TCM-inspired delivery systems—including self-assembled nanomedicines, cell-membrane–coated nanoparticles, immunogenic nanovaccines, hydrogels, and microneedle patches—shows that herb-derived compounds can be embedded in modern formulations that concentrate their activity within tumors, draining lymph nodes, or post-surgical beds [9, 32–36]. Many of these platforms combine a cytotoxic or ferroptosis/ICD-inducing core with immunomodulatory components, thereby amplifying ICD and cyclic GMP–AMP synthase (cGAS)–stimulator of interferon genes (STING) or inflammasome signaling while limiting collateral injury to non-malignant tissues [18, 22, 37, 38].

Against this background, the present review has three aims. First, we summarize how metabolic reprogramming of the TME influences key immune populations, focusing on glycolysis and lactate, mitochondrial integrity and ferroptosis/ICD, lipid–bile-acid circuits, and DC/T-cell bioenergetics [5, 39, 40]. Second, we organize TCM-derived interventions along these four mechanistic axes and examine their impact on tumor–immune metabolism at the level of individual compounds, formulas, and delivery platforms [41–44]. Third, we integrate these axes with core TCM doctrines to propose a framework for phenotype-guided, biomarker-informed co-therapy that remains accessible to clinicians and researchers without formal TCM training [1, 45, 46]. By reframing TCM through an immunometabolic lens, we aim to move beyond generic notions of “immune boosting” toward a mechanism-based rationale for combining herb-derived interventions with immune checkpoint inhibitors, targeted agents, and chemotherapy [47–49].

To reduce subjectivity in this narrative classification, we assigned each TCM-derived intervention to a primary immunometabolic axis according to four criteria: (i) the dominant metabolic process directly affected by the intervention, such as glycolytic flux, lactate export, mitochondrial damage, ferroptosis, lipid/bile-acid metabolism, or immune-cell bioenergetics; (ii) the major immune-functional bottleneck relieved by the intervention, including impaired antigen presentation, T-cell exhaustion, suppressive myeloid polarization, or insufficient immunogenic tumor death; (iii) the experimental readouts reported in the original studies, such as LDHA, PKM2, lactate, HIF-1α, ROS, GPX4, calreticulin exposure, high mobility group box 1 (HMGB1) release, bile-acid profiles, TAM/MDSC phenotypes, DC maturation, CD8⁺ T-cell infiltration, and interferon-gamma (IFN-γ) production; and (iv) the level of supporting evidence, ranging from in vitro assays and animal models to omics-based analyses, clinical cohorts, and interventional trials. When a compound or formula affected more than one process, it was classified by its most directly supported primary axis, while secondary axes were indicated as mechanistically coupled effects. Accordingly, the four-axis framework is intended to organize heterogeneous evidence into a mechanistically interpretable structure, not to imply that each TCM intervention acts through a single pathway or that current evidence is sufficient for clinical decision-making.

## Metabolic reprogramming in the TME: implications for immunity

Solid tumors profoundly reshape nutrient delivery, metabolite clearance, and oxygen tension. These changes not only sustain malignant proliferation but also redefine the metabolic context in which DCs, T cells, TAMs, MDSCs, and stromal populations operate [2, 11, 39, 48]. Functionally, the TME can be viewed as an ecosystem organized around a limited number of metabolic “axes” that repeatedly steer immune differentiation, survival, and effector function.

In this review, four interconnected aspects of tumor metabolism are particularly relevant: (i) glycolytic dominance and the “lactate economy”; (ii) lipid and bile-acid signaling in stromal myeloid niches; (iii) amino-acid metabolism, with an emphasis on tryptophan–AhR pathways; and (iv) mitochondrial fitness, redox homeostasis, and immunogenic forms of tumor cell death. The subsections below outline each axis as a framework for understanding how TCM-derived interventions intersect with these circuits.

### Glycolytic dominance and the lactate economy

Many cancers exhibit pronounced aerobic glycolysis (the “Warburg effect”), consuming large quantities of glucose and exporting lactate even under normoxic conditions [7, 8]. This glycolytic predominance imposes several layers of immune suppression.

First, tumor and stromal cells monopolize glucose, restricting access for infiltrating DCs and effector T cells and limiting the short glycolytic bursts required for antigen processing, cytokine secretion, and clonal expansion [9, 10]. Second, continuous lactate export acidifies the TME, directly inhibiting cytotoxic T-lymphocyte proliferation and function, while favoring regulatory T cells and tolerogenic myeloid phenotypes [2, 5, 10]. Third, glycolytic intermediates fuel biosynthesis and stabilize hypoxia-inducible factor 1-alpha (HIF-1α), which in turn upregulates glycolytic enzymes, angiogenic mediators, and immune checkpoints, further consolidating an immunosuppressive state [4, 18, 50].

Clinically, high glycolytic signatures and elevated lactate dehydrogenase (LDH) levels correlate with reduced immune infiltration, poor responsiveness to immune checkpoint inhibitors, and unfavorable prognosis across multiple tumor types [51, 52]. From an immunometabolic perspective, this axis therefore exerts a dual impact: it sustains tumor growth while simultaneously depriving immune cells of fuel and exposing them to toxic concentrations of lactate [2, 50, 53].

Conceptually, the resulting “lactate economy”—excess production combined with inefficient clearance—represents a major barrier that any immunotherapy must overcome. Several TCM-based approaches, including Gegen Qinlian decoction (GQD), Astragalus- and ginseng-containing prescriptions, and defined small molecules, have been reported to reduce tumor glycolytic flux and lactate accumulation while preserving or even supporting immune cell glycolysis [41–43, 47, 54, 55]. These findings foreshadow the Axis I interventions discussed later in this review.

### Lipid metabolism and bile-acid signaling: stromal myeloid programming

Tumors also remodel lipid metabolism at both systemic and local levels. Cancer cells upregulate de novo lipid synthesis, fatty-acid uptake, and β-oxidation to support membrane biogenesis, energy generation, and redox buffering, while host metabolism adapts with altered circulating lipid and lipoprotein profiles [3, 18, 56]. Within the TME, these changes are mirrored by stromal and immune cells: TAMs and MDSCs frequently accumulate cholesterol and triacylglycerols, rely more heavily on oxidative phosphorylation (OXPHOS), and adopt anti-inflammatory, antigen-presentation–poor programs [11, 12, 48].

Bile acids introduce an additional layer of regulation, particularly along the gut–liver axis. Disturbed bile-acid pools and signaling through receptors such as farnesoid X receptor and Takeda G protein–coupled receptor 5 influence Kupffer cells, liver-resident DCs, TAMs, and MDSCs, typically promoting immunosuppressive phenotypes and facilitating tumor progression and metastasis [3, 13, 56]. These lipid–bile-acid circuits can weaken NKT-cell surveillance and blunt responses to systemic therapies, especially in hepatocellular carcinoma and colorectal cancer with prominent hepatic involvement.

Together, dysregulated lipid metabolism and bile-acid signaling help establish metabolically privileged myeloid niches that shield tumor cells and reprogram systemic immunity. As discussed later, several TCM formulas—notably Jianpi Jiedu and Jianpi Huayu decoctions, Xiayuxue decoction (XYXD)-like prescriptions, Compound Kushen Injection, and Compound Fuling Granule—appear to act on these axes, repolarizing TAMs, reducing MDSC burden, and modulating the gut–liver environment [57–62]. These features provide the mechanistic foundation for Axis III.

### Amino-acid economy and tryptophan–AhR signaling

Beyond glucose and lipids, amino acids provide a third layer of metabolic regulation within the TME. Tumor and stromal cells compete with lymphocytes for arginine, glutamine, and branched-chain amino acids, while enzymes such as arginase and glutaminase determine local availability and thereby influence T-cell proliferation, differentiation, and effector function [11, 46]. In nutrient-poor niches, these shifts can selectively disadvantage highly active immune populations.

Among these pathways, tryptophan metabolism has emerged as particularly important for tumor immunity. Indoleamine 2,3-dioxygenase 1 (IDO1) and related enzymes degrade tryptophan into kynurenine, which acts on AhR expressed by T cells and myeloid cells. In the TME, the combination of tryptophan depletion and accumulating kynurenine promotes regulatory T-cell expansion, drives tolerogenic DC and TAM phenotypes, and suppresses cytotoxic responses, thereby linking amino-acid metabolism directly to immune escape [12, 46, 63].

Although mechanistic evidence remains incomplete, several TCM prescriptions—especially spleen-fortifying and detoxifying formulas such as Jianpi Jiedu and Jianpi Huayu decoctions—have been reported to modulate tryptophan–AhR signaling and associated inflammatory circuits in gastrointestinal tumors [48, 60, 61]. These observations suggest that amino-acid economy, and tryptophan metabolism in particular, represents an additional dimension through which TCM interventions may influence tumor–immune crosstalk.

### Mitochondrial fitness, redox balance and immunogenic tumor death

Effective antitumor immunity requires that tumor cells are sufficiently vulnerable to “dangerous” modes of death, while immune cells retain resilience in the face of metabolic and oxidative stress. On the tumor side, mitochondrial dysfunction, reactive oxygen species (ROS) generation, lipid peroxidation, and iron handling together determine whether cell death is immunogenic or immunologically silent [18, 22, 64].

Ferroptosis—a regulated, iron-dependent form of cell death driven by lipid peroxidation—has attracted particular attention. Ferroptotic tumor cells can release damage-associated molecular patterns (DAMPs), expose oxidized lipids, and stimulate DC activation and cross-presentation, thereby enhancing CD8⁺ T-cell responses and synergizing with checkpoint blockade [14–16, 34]. However, sublethal ferroptotic stress or uncontrolled necrosis may instead generate immunosuppressive signals, fuel chronic inflammation, or contribute to effector-cell dysfunction [5, 39, 53].

In parallel, mitochondrial integrity and redox balance in DCs and T cells are essential for durable immune responses. Excess ROS, persistent mitochondrial damage, or misdirected ferroptosis in immune compartments can compromise antigen presentation, promote T-cell exhaustion, and disturb immune homeostasis [17, 18, 64]. These considerations underpin the design of ferroptosis- and ICD-oriented nanomedicines and hydrogels that localize pro-death stimuli to tumor tissue while sparing non-malignant immune cells [22, 35, 65].

A number of these platforms incorporate TCM-derived components. Systems built on Schottky heterojunction photocatalysts, covalent organic frameworks, dextran–chitosan hydrogels, microneedles, or vesicle-like carriers can enhance ICD or ferroptosis, amplify cGAS–STING or inflammasome signaling, and increase tumor antigen release in a controllable manner [16, 22]. Preclinical work indicates that such interventions boost DC cross-priming and CD8⁺ T-cell activity, and may help overcome resistance to checkpoint inhibitors in otherwise “cold” tumors [34, 37, 66].

On the immune side, DCs and T cells require preserved mitochondrial respiration and the ability to switch between glycolysis and OXPHOS to sustain activation, migration, and memory formation [39, 50]. Chronic hypoxia, nutrient deprivation, and oxidative stress within the TME progressively erode this flexibility and promote T-cell exhaustion [5, 6, 10]. Several TCM-derived interventions—including Astragalus-based prescriptions, Huaier preparations, and Ganoderma fractions—have been reported to support mitochondrial function, enhance antioxidant defenses, and partially restore immune-cell metabolic fitness in preclinical models [67–71]. These properties lie at the heart of Axis II and Axis IV.

### Spatial–temporal heterogeneity and systems-level readouts

Tumor metabolic states are heterogeneous across both space and time. Well-perfused peripheral regions may differ sharply from hypoxic, necrotic cores in oxygen and nutrient availability, lactate concentration, lipid utilization, bile-acid exposure, and redox status [1, 6, 13, 72]. Immune infiltration and stromal composition vary across these zones as well, so cells residing in distinct niches experience different metabolic pressures and can respond quite differently to a given intervention [10, 73–75].

This heterogeneity implies that neither targeted metabolic inhibitors nor TCM formulas act uniformly throughout a tumor. Their impact depends on where and when they are delivered, and on the extent to which they modulate each metabolic axis. Multi-omic profiling, functional metabolic imaging, and spatial transcriptomics will be essential to capture these patterns and to design axis-based treatment strategies that explicitly accommodate intra-tumor and inter-patient diversity rather than relying on one-size-fits-all regimens [39, 76, 77].

## TCM as an immunometabolic co-therapy

TCM is often summarized as “supporting vital Qi and dispelling pathogenic factors,” a phrase that already implies reciprocal action on both host and tumor. Rather than targeting a single molecule, classical prescriptions are assembled to correct broader Zheng (“patterns”) that integrate metabolism, inflammation, barrier function, and emotional state. From an immunometabolic standpoint, this means that many formulas concurrently influence glycolysis and lactate handling, mitochondrial fitness and ICD/ferroptosis, lipid and bile-acid circuits in myeloid niches, and the bioenergetic resilience of DCs, T cells, and innate lymphocytes [1, 47–49]. Such multi-axis modulation is difficult to emulate with single-target inhibitors.

Mechanistic and omics-based work on formulas such as Xihuang Pill, Kejinyan decoction, Jianpi Jiedu and Jianpi Huayu prescriptions, together with preparations like Compound Kushen Injection, illustrates this systems-level logic [21, 29, 30, 58]. Across diverse models, these interventions can lower tumor glycolytic pressure, modulate lipid and bile-acid metabolism, reprogram tumor-infiltrating myeloid precursors, and enhance DC/T-cell activation in parallel [57, 60, 61]. More recently, tonifying polysaccharides and multi-herb decoctions have been shown to reshape tumor-infiltrating myeloid and antigen-presenting cell networks in situ, providing a direct mechanistic bridge between ethnopharmacological practice and contemporary tumor immunology [40, 48, 78, 79]. Conceptually, this aligns with the TCM principle of “treating the root and branch together,” i.e. rebalancing a disturbed system rather than simply “hitting” one node.

To make this complexity manageable, we organize TCM-derived interventions along four recurrent immunometabolic axes. Axis I focuses on glycolysis and lactate, describing how modulation of tumor glycolytic flux and lactic acid load influences the immune microenvironment. Axis II centers on mitochondrial integrity and ferroptosis/ICD, highlighting the need to elicit “dangerous” forms of tumor cell death while preserving immune bioenergetics. Axis III covers lipid and bile-acid reprogramming of myeloid niches, especially along the gut–liver axis where dysregulated metabolites skew macrophage and MDSC phenotypes. Axis IV encompasses DC/T-cell metabolic fitness, emphasizing the importance of maintaining immune-cell flexibility, survival, and effector function under metabolic stress [3, 39, 64].

Axis I–IV are presented as separable modules for clarity, but they are not mutually exclusive. Each axis represents a dominant immunometabolic pressure node with characteristic biomarkers, functional bottlenecks, and actionable levers. Overlap reflects predictable crosstalk that can be mapped to defined coupling nodes. For example, lactate-driven acid stress and HIF-1α signaling (Axis I) converge on redox imbalance and mitochondrial dysfunction (Axis II), while lipid peroxidation and ferroptosis-associated cues connect Axis III to Axis II. Nutrient competition links Axis I to immune-cell metabolic fitness and exhaustion programs (Axis IV), and bile-acid/AhR signaling provides an interface between Axis III and Axis IV. Accordingly, we define the primary axis as the axis that most directly constrains antigen presentation or effector function in a given setting, and the secondary axis as coupled modules that amplify or sustain the phenotype. Many TCM interventions therefore map to a primary axis with secondary effects: GQD dampens tumor glycolysis while normalizing myeloid trafficking; Astragalus- and Ganoderma-derived polysaccharides alleviate lactate stress and support DC/T-cell bioenergetics; Jianpi Jiedu/Jianpi Huayu prescriptions reshape lipid–bile-acid metabolism and tryptophan–AhR signaling; and monomers such as ginsenosides, emodin, and ursolic acid can trigger ICD/ferroptosis while preserving immune metabolic competence [48, 80, 81].

In this review, the term “primary axis” does not imply exclusivity. Rather, it denotes the axis for which the most direct mechanistic evidence is available. For example, a formula was considered Axis I-dominant when studies primarily measured glycolytic enzymes, glucose uptake, lactate production, or acidification together with immune restoration. It was considered Axis II-dominant when the central evidence involved mitochondrial injury, ROS accumulation, ferroptosis, ICD markers, and subsequent DC or T-cell activation. Axis III was assigned when lipid metabolism, bile-acid signaling, microbiota–liver interactions, TAM polarization, or MDSC remodeling represented the main mechanistic focus. Axis IV was assigned when the strongest evidence concerned DC maturation, antigen presentation, T-cell metabolic fitness, Th1 polarization, or effector/memory T-cell responses. This approach allows multicomponent TCM interventions to be discussed without forcing them into mutually exclusive categories.

A second organizing layer concerns drug delivery. Classical decoctions and pills act systemically and are subject to first-pass metabolism and microbiota-mediated transformation. In contrast, modern TCM-inspired platforms—including nanoparticles, hydrogels, biomimetic carriers, and in situ vaccines—are engineered to concentrate active components in hypoxic tumor cores, draining lymph nodes, or post-surgical beds [9, 37, 82]. Platelet-membrane–camouflaged or cell-membrane–coated nanomedicines have been used to deplete glutathione, amplify radiotherapy-induced ICD, and enhance checkpoint blockade [33, 35, 37, 64]. Nanoparticles encapsulating Astragalus polysaccharides, ginsenoside Rg3 (Rg3), or other monomers can be combined with focused ultrasound, phototherapy, or chemotherapy to synchronize ICD induction with immune activation [34, 81, 83, 84].

Taken together, these patterns support viewing TCM as an immunometabolic co-therapy rather than merely supportive or adjunctive care. By rebalancing glucose, lipid, and redox pathways; promoting ICD/ferroptosis; and stabilizing DC/T-cell metabolic fitness, herb-derived interventions may help convert “cold” tumors into “hot” lesions, deepen responses to checkpoint inhibitors, adoptive T-cell therapy, and CAR-T cells, and potentially mitigate treatment-related toxicity.

## Herb-derived compounds targeting key metabolic pathways

**Fig. 1:**
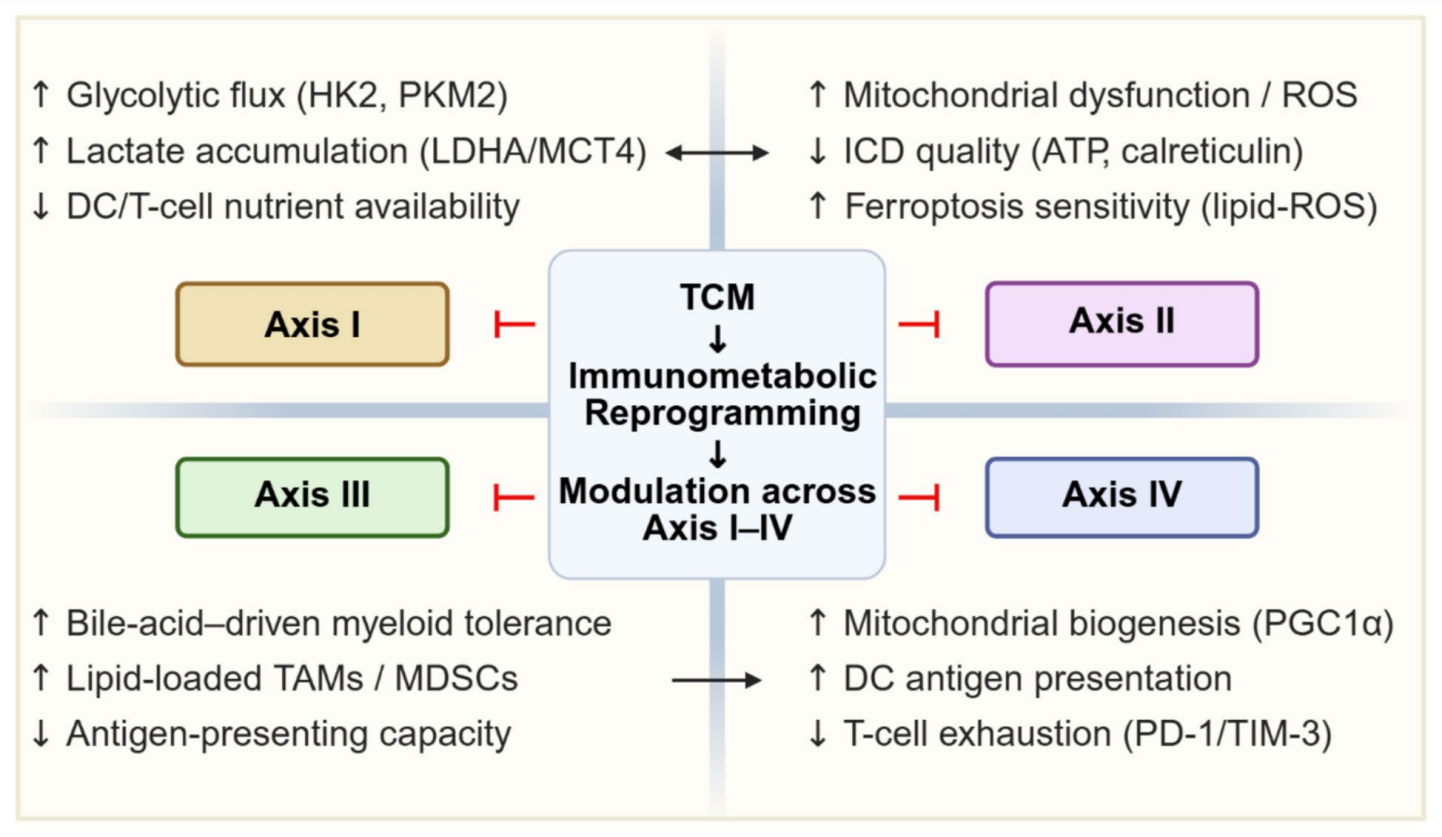
Four immunometabolic axes linking TCM-derived interventions to tumor–immune remodeling. The proposed framework organizes TCM-derived compounds, formulas, and delivery systems into four interconnected immunometabolic axes: Axis I, glycolysis–lactate metabolism; Axis II, mitochondrial integrity and ferroptosis/ ICD; Axis III, lipid–bile-acid–centered myeloid niches; and Axis IV, DC/T-cell metabolic fitness. Each intervention is assigned to a primary axis according to its dominant metabolic target, immune-functional bottleneck, experimental readouts, and level of supporting evidence. Secondary axes indicate predictable crosstalk among lactate stress, redox imbalance, lipid remodeling, ferroptosis, antigen presentation, and T-cell exhaustion

Traditional formulas and isolated compounds from TCM provide concrete examples of how herbal interventions engage the four immunometabolic axes described above. In this section, representative agents and prescriptions with relatively well-defined mechanisms and documented immune effects are grouped according to their predominant impact on glycolysis and lactate (Axis I), mitochondrial integrity and ICD/ferroptosis (Axis II), lipid–bile-acid–centered myeloid niches (Axis III), and DC/T-cell metabolic fitness (Axis IV) (Fig. 1; Table 1). Because these axes are interconnected, we classify each intervention by its predominant (primary) axis and summarize recurrent secondary effects through predictable axis-to-axis coupling.

**Table 1:** Representative TCM-derived interventions and their dominant immunometabolic axis

Axis	TCM intervention	Main metabolic targets	Major immune effects	Evidence level and key evidence
Axis I	GQD	↓ PKM2; ↓ lactate; ↓ glycolytic flux	↑ Myeloid/T-cell trafficking; ↑ antigen presentation	L1; GI tumors; Met + Imm
Axis I	Astragalus-containing prescriptions; Astragalus polysaccharide	PI3K–Akt–mTOR/HIF-1α; ↓ tumor glycolysis	↑ DC/T-cell flexibility; ↑ Th1/CD8⁺	L1–L2; solid/GI; Met + Imm
Axis I	Quxie Capsule	Reshapes intestinal/tumor milieu; ↓ lactate	Restores immune surveillance in CRC	L1–L2; CRC; Met + Imm
Axis II	Ginsenoside Rg3; Rg3 nanomedicines	↑ mito stress; apoptosis/ferroptosis; ↓ PD-L1	↑ DC cross-priming; ↑ CD8⁺; ↑ ICI response	L1; solid tumors; ICD/Ferro + Imm; + ICB
Axis II	Celastrol/gambogic acid nanoplatforms	↑ ROS; ↑ ICD; mito targeting	↑ DAMPs; ↑ cGAS–STING; ↑ antitumor T cells	L1; solid tumors; ICD + STING; ± Combo
Axis II	Fe₃O₄ hybrids; COF hydrogels	↑ ferroptosis; ↑ lipid peroxidation	↑ DC priming; ↑ checkpoint efficacy	L1; HCC/peritoneal; Ferro + Imm; + ICB
Axis III	Jianpi Jiedu; Jianpi Huayu decoctions	BA/lipid handling; microbiota reshaping	↓ MDSC/TAM; ↑ DC/T-cell function	L1–L2; GI/HCC; BA/Lipid + Myeloid
Axis III	Compound Kushen Injection	TGF-β/Smad; hepatic lipid-inflammation	Rebalances hepatic myeloid niches	L1–L2; HCC; Met + Myeloid
Axis III	Stigmasterol; emodin; ginsenosides (selected)	↓ macrophage lipid overload; ↓ inflammation	TAM repolarization	L0–L1; macrophage ± tumor; Lipid + TAM
Axis IV	Astragalus regimens; Hochu-ekki-to	↑ mito respiration; antioxidant defence	↑ tolerance; ↑ CD8⁺/Th1	L1–L2; solid; Fitness + Imm
Axis IV	Huaier; Ganoderma polysaccharides; SQYC	↑ DC maturation/costimulation	↑ IFN-γ⁺ T cells; ↑ priming/effector	L1–L2; solid; DC + T
Axis IV	Ginseng vesicle-like nanoparticles; herbal microneedles	LN delivery; adjuvant effect	↑ DC activation; ↑ antigen-specific T cells	L1; vaccine models; LN/DC + T; + Vax

*BA* bile acid, *COF* covalent organic framework, *CRC* colorectal cancer, *DC* dendritic cell, *Ferro* ferroptosis, *GI* gastrointestinal, *GQD* Gegen Qinlian decoction, *HCC* hepatocellular carcinoma, *ICD* immunogenic cell death, *ICB/ICI* immune checkpoint blockade/inhibitor, *LN* lymph node, *Met/Imm* metabolic/immune readouts, *MDSC* myeloid-derived suppressor cell, *mito* mitochondrial, *ROS* reactive oxygen species, *SQYC* Shenqi Yiqi Capsule, *TAM* tumor-associated macrophage, *TCM* Traditional Chinese Medicine, *Vax* vaccination. Evidence levels: L0, mechanistic in vitro/ex vivo; L1, in vivo preclinical; L2, clinical observational/real-world; L3, interventional clinical study. Tags: Met + Imm, metabolic and immune endpoints; BA/Lipid + Myeloid, bile-acid/lipid handling and myeloid-niche endpoints; ICD/Ferro + Imm, ICD/ferroptosis and immune endpoints; + ICB/ + Vax, combination context

To avoid an indiscriminate catalogue of herbal agents, we prioritized interventions that met at least two of the following criteria: (i) reported effects on defined metabolic readouts; (ii) parallel assessment of immune-cell phenotypes or immune-functional endpoints; (iii) validation in animal tumor models or patient-derived samples; (iv) support from transcriptomic, metabolomic, microbiome, or spatial profiling; and (v) relevance to clinically used formulas, patent medicines, or modern delivery systems. Interventions supported only by isolated in vitro cytotoxicity data are mentioned cautiously and are not treated as clinically validated immunometabolic modulators. Therefore, the following subsections focus on recurring mechanistic patterns and translational bottlenecks rather than attempting to exhaustively list all reported herbal compounds.

### Axis I—glycolysis and lactate: lowering acid load and restoring antigen presentation

#### Enzymatic throttling of tumor glycolysis

Several herb-derived small molecules act directly or indirectly on key glycolytic enzymes and transporters. Typically, these compounds reduce glucose uptake, suppress the expression or activity of glycolytic kinases, and limit lactate export, thereby attenuating Warburg-like metabolism and lowering the acid burden in the TME. By softening glycolytic pressure rather than abolishing energy production, they can restore sensitivity to chemotherapy and targeted agents in highly glycolytic, metabolically stressed tumors [41, 80, 85].

At the microenvironmental level, Quxie Capsule illustrates this principle in colitis-associated colorectal cancer. By reshaping the intestinal milieu and altering tumor cell metabolism, Quxie Capsule decreases glycolytic signatures and improves local immune surveillance. Its effects extend beyond direct tumor inhibition to stromal and immune compartments, reconfiguring tumor–gut–immune crosstalk rather than behaving as a non-selective glycolytic poison [85, 86].

At the level of regulatory signaling, several TCM-inspired molecules modulate pathways such as PI3K–AKT–mTOR, HIF-1α, and STAT3, which coordinate glucose metabolism with immune-evasion programs. By tuning these nodes, herb-derived monomers can reduce programmed death-ligand 1 (PD-L1) expression, improve antigen processing, and enhance DC and T-cell activity even in tumors that remain partially glycolytic, thereby helping to rebalance metabolic and immunological responses [4, 47].

Conceptually, these interventions function more like metabolic “brakes” than complete shutdown switches. Excessive inhibition of glycolysis may damage proliferating immune cells, whereas careful throttling of tumor glycolysis and lactate release relieves DC and T-cell suppression while preserving their capacity for brief glycolytic bursts during activation. TCM-derived small molecules that modestly dampen glycolytic flux and rewire stress responses thus offer a nuanced alternative to aggressive, systemic glycolytic blockade in highly glycolytic, immunosuppressive microenvironments [41, 80, 85, 87].

#### Prescriptions that rebalance glycolysis and myeloid/T-cell traffic

GQD is a clear example of a multi-herb prescription acting on Axis I. In colorectal and gastric cancer models, GQD inhibits tumor proliferation, downregulates glycolytic enzymes such as PKM2, reduces lactate accumulation, and partially normalizes the composition of the TME. These changes are accompanied by improved trafficking and activation of myeloid and T-cell populations, suggesting that GQD lowers the “glycolytic wall” that excludes effector cells while directly influencing PKM2 and related regulators [11, 42, 54].

Related spleen-fortifying formulas, including Jianpi Jiedu and Jianpi Huayu decoctions, also suppress tumor growth in gastrointestinal and hepatobiliary models. They appear to combine moderate suppression of tumor glycolysis with myeloid reprogramming and enhancement of barrier function along the gut–liver axis, thereby easing immunosuppressive pressure in glycolysis-high tumors characterized by strong lactate production and immune exclusion [7, 10, 51].

Astragalus-based prescriptions constitute another important class of Axis I modulators. Astragalus polysaccharide and Astragalus-containing decoctions reduce tumor glycolytic activity in several preclinical models while supporting systemic and local immune function. By decreasing lactate accumulation and preserving DC and T-cell glycolytic flexibility, these formulas create a therapeutic window during which vaccines, checkpoint inhibitors, or cytotoxic agents can act more effectively, linking traditional “Qi-tonifying” strategies to a modern immunometabolic rationale [49, 81].

Taken together, Axis I–oriented TCM interventions exemplify a strategy of “lowering the metabolic ceiling” rather than turning glycolysis off. By gently reducing tumor glycolysis and lactate export while stabilizing immune-cell energy supply, these compounds and formulas help restore antigen presentation, improve T-cell infiltration, and sensitize tumors to chemoimmunotherapy.

### Axis II—mitochondrial integrity and immunogenic tumor death (ferroptosis/ICD)

For Axis II, the key issue is not only whether tumor cells die, but how they die and whether that process meaningfully engages antitumor immunity. TCM-inspired strategies along this axis aim to push tumors toward immunogenic modes of death—particularly ICD and ferroptosis—while maintaining mitochondrial function and redox balance in immune cells. The ideal outcome is a combination of selective tumor lethality and preserved immune bioenergetics [18, 34].

One group of TCM-based approaches centers on mitochondria-targeted systems. Herb-derived components are incorporated into nanoplatforms, hydrogels, or in situ vaccines that localize ROS bursts, mitochondrial damage, and ICD to tumor cells. By confining oxidative stress and mitochondrial collapse to malignant tissue, these systems increase DAMP release, antigen exposure, and type I interferon production, yet limit collateral injury in surrounding normal cells [16, 33, 37, 65]. Examples include Schottky heterojunction photocatalysts, covalent organic framework–based composites, and dextran–chitosan hydrogels that co-deliver herbal monomers with photothermal, photodynamic, or chemotherapeutic cues [16, 22, 34, 35].

A second group of interventions emphasizes ferroptosis-enhanced immunotherapy. Iron-containing nanohybrids and TCM-derived constructs that sensitize cancer cells to lipid peroxidation have been shown to amplify ICD and improve the efficacy of immune checkpoint blockade in preclinical models [17, 34, 88]. These designs often combine iron catalysis with glutathione depletion, GPX4 inhibition, and immunostimulatory signals, producing a controlled wave of ferroptotic death that is tightly linked to DC activation, STING or inflammasome signaling, and improved antigen presentation [17, 35, 88, 89]. Dihydroartemisinin, a derivative of artemisinin, has also been reported to induce ferroptosis in pancreatic cancer cells through regulation of survival prediction-related genes, further supporting the relevance of ferroptosis-oriented herb-derived interventions within Axis II [90].

In parallel, several TCM-derived small molecules modulate mitochondrial and redox pathways within immune cells. By supporting OXPHOS, limiting excessive ROS accumulation, and preventing maladaptive ferroptotic responses in DCs and T cells, these agents help preserve antigen-processing capacity, cytokine production, and memory formation even in metabolically stressed tumors [64, 67, 71, 87, 89]. This dual action—pushing tumor cells toward immunogenic death while stabilizing immune bioenergetics—is a defining feature of Axis II–oriented strategies.

Across these ICD- and ferroptosis-focused designs, a common theme emerges: TCM-derived compounds and formulations are used not simply as cytotoxic agents, but as tools to choreograph where and how tumor cells die. When successful, they convert local cytotoxic events into zones rich in neoantigen presentation and productive inflammation, thereby linking cell death to durable immune control.

### Axis III—lipid and bile-acid reprogramming of myeloid niches

Axis III focuses on how lipid and bile-acid metabolism shape myeloid niches and, through them, systemic immunity. TAMs, Kupffer cells, and MDSCs within the TME frequently display altered lipid uptake, storage, and oxidation, features that stabilize immunosuppressive phenotypes and limit antigen presentation. Disordered bile-acid pools and receptor signaling along the gut–liver axis further reinforce these myeloid programs, particularly in hepatocellular carcinoma and colorectal cancer with hepatic involvement [3, 91].

Several individual compounds illustrate how TCM can act on this axis. Stigmasterol, ursolic acid, and emodin have been reported to normalize lipid handling, attenuate inflammatory cascades, and promote antitumor TAM polarization in preclinical models [56, 58, 62]. These molecules frequently appear as active components within broader TCM regimens such as Compound Kushen Injection and Compound Fuling Granule, where they contribute to reshaping myeloid metabolism and function [58].

Spleen-fortifying and detoxifying formulas extend these mechanisms within clinically recognizable Zheng patterns. Jianpi Jiedu and Jianpi Huayu decoctions, for instance, adjust bile-acid and lipid profiles, remodel gut microbiota, and reduce expansion of MDSCs and tolerogenic TAMs in gastrointestinal and hepatobiliary tumors [59–61, 92]. Their actions span intestinal barrier integrity, hepatic metabolic homeostasis, and tumor-infiltrating myeloid cell states—precisely the domains emphasized in “spleen deficiency,” “damp-heat,” and related diagnostic categories.

Bioinformatic analyses reinforce these mechanistic observations. Network pharmacology and transcriptomic studies reveal that Axis III–oriented formulas converge on gene modules implicated in lipid metabolism, bile-acid transport, and myeloid differentiation not only in liver cancer, but also in other tumors with strong myeloid components, such as pancreatic and colorectal cancer [28, 62, 75, 78]. Collectively, Axis III interventions highlight the potential of TCM to “reopen” lipid-driven myeloid bottlenecks and create conditions conducive to effective antitumor immunity and durable responses to systemic therapy.

### Axis IV—restoring DCs and T-cell metabolic fitness

The fourth axis centers on the metabolic state of immune cells themselves. Effective antitumor responses require DCs that can maintain antigen processing and cross-presentation, and T cells that can withstand metabolic stress without sliding into exhaustion. In the TME, chronic hypoxia, nutrient competition, lactate overload, and oxidative stress erode this flexibility, pushing immune cells toward dysfunctional states that undermine long-term control [5, 82, 93].

TCM-derived interventions can support DC and T-cell metabolism through several routes. Tonifying polysaccharides, including Astragalus and Ganoderma fractions, enhance DC maturation, promote costimulatory molecule expression, and sustain glycolytic bursts needed for antigen presentation [45, 83, 97–99]. Huaier and Shenqi Yiqi Capsule (SQYC) have been reported to increase DC numbers, improve cytokine production, and boost Th1-polarized responses in patients receiving chemotherapy or chemoimmunotherapy, suggesting that they help preserve DC function under therapeutic stress [83, 97, 99, 100].

These immune-centered effects extend to more complex formulas that combine Qi-tonifying and detoxifying components. Such prescriptions often improve lymphocyte counts, enhance vaccine responses, and alleviate symptoms of fatigue and cachexia in clinical settings, consistent with their traditional classification as “supporting vital Qi” in debilitated patients [95–99]. Mechanistically, they are linked to improved mitochondrial respiration, better redox buffering, and restored metabolic flexibility in DCs and T cells.

On the T-cell side, ginsenoside Rg3 and related ginsenosides exemplify how tumor-centered and T-cell-centered actions can be aligned. By reducing tumor PD-L1 expression, inducing ICD or ferroptosis, and supporting mitochondrial function in T cells, these agents have been shown to enhance CD8⁺ T-cell proliferation, effector function, and memory formation in preclinical models [81–83, 93]. In principle, such interventions can be layered onto checkpoint blockade or CAR-T therapies to help rescue exhausted T cells in glycolytic, acidic niches.

In summary, Axis IV captures TCM’s capacity to stabilize immune-cell metabolism in hostile microenvironments. By reinforcing DC and T-cell bioenergetics, these interventions can transform transient bursts of antigen release—whether from chemotherapy, radiotherapy, or ICD/ferroptosis-inducing nanomedicines—into sustained, T cell–mediated immune surveillance and improved responses to checkpoint inhibitors, vaccines, and adoptive cell therapies.

Across Axis I–IV, the strongest evidence currently comes from interventions that combine metabolic readouts with immune-functional validation, particularly in animal tumor models or omics-supported systems. By contrast, studies limited to changes in tumor-cell viability or isolated signaling proteins provide weaker support for true immunometabolic remodeling. This distinction is important because many TCM-derived agents exert broad pharmacological effects, and only a subset have been shown to link metabolic modulation with measurable changes in antigen presentation, myeloid polarization, T-cell function, or therapeutic response.

## Modulation of immune cells via metabolic reprogramming by TCM

**Fig. 2:**
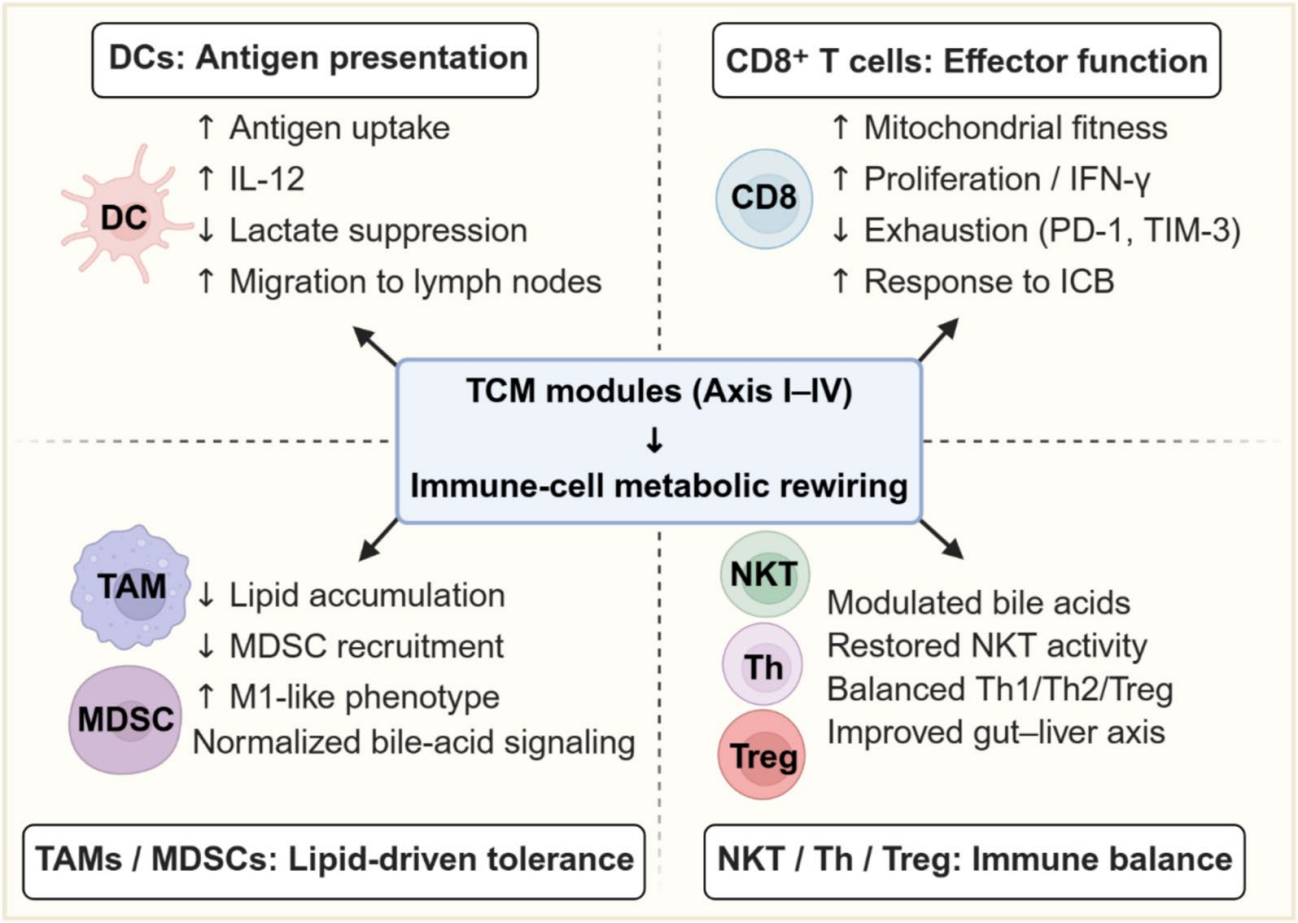
Immune-cell populations regulated by TCM-mediated immunometabolic remodeling. TCM-derived immunometabolic interventions may affect multiple immune compartments within the tumor microenvironment. Axis I mainly relieves lactate-driven suppression of DCs and CD8⁺ T cells; Axis II promotes immunogenic tumor death and dendritic-cell cross-priming while preserving immune-cell redox balance; Axis III reprograms TAMs, MDSCs, Kupffer cells, and gut–liver myeloid niches through lipid and bile-acid pathways; and Axis IV directly supports dendritic-cell maturation, antigen presentation, Th1 polarization, and T-cell metabolic fitness. The figure emphasizes functional interactions rather than one-to-one mapping between individual formulas and single immune subsets

TCM-derived interventions influence multiple immune lineages through their combined effects on the four axes described above. Rather than acting on single receptors or pathways, classical formulas and herb-derived monomers remodel the metabolic context in which DCs, T cells, TAMs, MDSCs, NK, and NKT cells operate. In this section, we summarize how TCM-driven metabolic reprogramming affects these key populations and how these changes help bridge classical TCM theory with contemporary tumor immunology (Fig. 2).

### DCs: guarding antigen presentation under metabolic stress

DCs sit at the interface between tumor metabolism and adaptive immunity. Chronic exposure to glucose competition, lactate accumulation, hypoxia, and lipid overload impairs DC maturation, reduces antigen processing and cross-presentation, and restricts migration to draining lymph nodes, ultimately weakening CD8⁺ T-cell priming [5, 10]. In such conditions, even a high neoantigen load may fail to elicit durable immunity if DCs cannot maintain metabolic flexibility and sufficient costimulatory capacity.

TCM-derived interventions support DC metabolism and function at several levels. Tonifying polysaccharides and formulas enriched in Astragalus and ginseng promote DC maturation, interleukin-12 (IL-12) production, and Th1-polarized responses in preclinical models [48, 78, 95]. Clinical data in non-small cell lung cancer indicate that combining ginseng-derived preparations with DC-based strategies improves the Th1/Th2 balance and enhances antitumor immunity, consistent with their traditional classification as “supporting vital Qi” [79]. Polysaccharides from Lonicera japonica and other herbs, when directed to lymph nodes, further augment DC activation and strengthen vaccine responses in metabolically stressed hosts [83, 97, 99].

Formulas such as SQYC, Huaier-containing regimens, and Ganoderma polysaccharides add further layers of DC support. These prescriptions enhance DC maturation, upregulate costimulatory molecules, and increase IFN-γ–producing T cells, thereby improving the quality of T-cell priming in hepatobiliary and gastrointestinal malignancies [25, 40, 48, 109]. Their effects are particularly relevant when chemotherapy or radiotherapy imposes additional metabolic pressure on antigen-presenting cells, as they help stabilize DC function during treatment [99, 100].

More targeted strategies incorporate TCM-derived agents into vaccines and biomimetic carriers. Ginseng-derived extracellular vesicle–like nanoparticles can act as both adjuvants and delivery vehicles to promote DC activation and antigen presentation [93, 94]. TCM-inspired nanomedicines that combine ICD inducers or ferroptosis triggers with DC-targeted adjuvants allow ICD signaling and DC activation to be co-engaged in a spatially controlled manner [35, 64, 82].

Taken together, available data support a model in which TCM prescriptions and monomers protect and re-energize DCs in metabolically hostile niches, preserving the initiating step of antitumor immunity. When combined with checkpoint inhibitors or conventional cytotoxic therapy, DC-supporting formulas and nanomedicines may help convert transient tumor antigen release into sustained T-cell–mediated control [48, 79, 95].

### CD8⁺ and Th1 T cells: countering exhaustion in a glycolytic, acidic niche

Effector T cells rely on glycolysis and robust mitochondrial function for clonal expansion, cytokine production, and cytotoxic activity. Within the TME, however, persistent antigen exposure, hypoxia, low glucose, and high lactate progressively drive T cells toward metabolic inflexibility, mitochondrial dysfunction, and an exhausted phenotype characterized by high checkpoint expression and impaired effector function [4, 5, 51]. These constraints are particularly evident in highly glycolytic tumors, where CD8⁺ T cells become confined to peripheral regions or lose cytotoxic capacity despite ongoing antigen recognition.

TCM-derived interventions may relieve these constraints from both the tumor and T-cell sides. Astragalus- and ginseng-based formulas reduce glycolytic pressure and lactate burden while enhancing T-cell proliferation and IFN-γ secretion, improving the quality of effector and memory pools [48, 49, 81]. Hochu-ekki-to and related prescriptions have been reported to reinforce T-cell responses in clinically stressed or immunosenescent hosts, in line with experimental evidence that polysaccharides can support mitochondrial respiration and antioxidant defenses in lymphocytes [25, 78].

Ginsenosides such as Rg3 exemplify how tumor-centered and T-cell–centered effects can be integrated. Rg3 and Rg3-loaded nanoparticles induce ferroptosis or apoptosis in tumor cells, downregulate PD-L1 expression, and enhance CD8⁺ T-cell infiltration and function in several solid tumors. In this setting, TCM-derived compounds not only provide additional tumor cell killing but also recondition the metabolic milieu experienced by T cells, facilitating sustained effector function and memory formation [40, 81, 84]. Collectively, these observations support incorporating Axis IV–oriented TCM interventions into regimens designed to rescue exhausted T cells within glycolytic, acidic niches.

### TAMs: rewiring lipid-driven tolerance

TAMs are key architects of the TME. In many cancers, they adopt a lipid-rich, OXPHOS-dependent, and anti-inflammatory phenotype that promotes angiogenesis, metastasis, and immune escape [3, 13, 56]. High levels of lipid uptake and storage, together with nuclear receptor activation and bile-acid signaling, stabilize suppressive TAM states and limit antigen presentation and T-cell recruitment.

TCM-derived strategies to reprogram TAMs frequently operate through Axis III. Jianpi Jiedu and Jianpi Huayu decoctions remodel metabolic and inflammatory circuits in colorectal and hepatocellular carcinoma, repolarizing TAMs away from protumor phenotypes and reducing MDSC burden [57, 60, 61, 91]. Mechanistic and network pharmacology studies indicate that these formulas simultaneously influence lipid and bile-acid metabolism, tryptophan–AhR signaling, and cytokine networks along the gut–liver axis, providing a systems-level explanation for their traditional indications in “spleen deficiency” and “damp-heat” patterns [11, 76].

Other preparations focus more specifically on liver-centered immunometabolism. Compound Kushen Injection improves liver function, inhibits fibrosis, and reduces hepatocarcinogenesis, in part by modulating TGF-β/Smad signaling and hepatic myeloid activation [58]. Stigmasterol, emodin, and certain ginsenosides attenuate lipid accumulation and inflammatory signaling in macrophages, suggesting that they may relieve the lipid overload that sustains tolerogenic TAM subsets [56, 62, 78].

Quxie Capsule and related approaches extend these concepts to colitis-associated cancer and other inflammation-driven malignancies. By reshaping the microbiota, modulating histone demethylases such as lysine-specific demethylase 4D (KDM4D), and normalizing lipid metabolism, these interventions dampen protumor inflammation and reduce the emergence of immunosuppressive myeloid niches [59, 92]. Together, Axis III–oriented TCM strategies suggest that “detoxifying” and “spleen-fortifying” prescriptions can be reinterpreted as efforts to reset TAM metabolism and restore a microenvironment permissive for effective antitumor immunity.

### MDSCs: eroding a metabolically protected barrier

MDSCs constitute a major cellular barrier to effective antitumor immunity. These immature myeloid cells expand in response to chronic inflammation, rely heavily on glycolysis, fatty-acid metabolism, and amino-acid catabolism, and suppress T-cell and NK-cell function through nutrient depletion, ROS production, and inhibitory cytokines. Their accumulation is particularly prominent in gastrointestinal and hepatobiliary tumors, where they cooperate with TAMs and regulatory T cells to establish a metabolically protected, immunosuppressive niche [11, 48, 57].

TCM-based combinations can erode this barrier through coordinated effects on metabolism and differentiation. Jianpi Jiedu and Jianpi Huayu decoctions decrease MDSC frequency and suppressive function in colorectal and liver cancer models while improving DC and T-cell activation [60, 61]. Formulas that reduce tumor glycolysis and lactate output, such as GQD, also limit MDSC recruitment to tumors and draining lymph nodes, further weakening this suppressive shield [42, 101]. By simultaneously restraining tumor-driven metabolic signals and promoting myeloid maturation, these prescriptions help re-establish a microenvironment in which effector cells can expand and function during therapy.

### NKT cells and tissue-restricted surveillance along the gut–liver axis

In hepatic tumors, bile-acid dysregulation, microbiota-derived metabolites, and chronic inflammation profoundly reshape the gut–liver axis. These changes perturb NKT-cell homeostasis, shifting cells away from cytotoxic surveillance toward regulatory or anergic states [12, 46]. Altered bile-acid pools and AhR ligands also reinforce suppressive macrophage phenotypes, creating a niche in which tumor cells can thrive despite the presence of abundant innate-like lymphocytes.

TCM interventions appear capable of partially restoring this tissue-restricted surveillance. Formulas used to “soothe the Liver and strengthen the Spleen,” including Jianpi Jiedu- and Jianpi Huayu-type prescriptions, modulate bile-acid profiles, intestinal barrier function, and liver inflammation in ways consistent with improved NKT-cell–mediated tumor control in experimental models [57, 60, 61]. Compound Kushen Injection and related liver-targeted therapies further rebalance hepatic immunometabolism and reduce fibrotic and protumor signaling, although the precise impact on individual NKT subsets still requires detailed mechanistic study [58].

Beyond TCM itself, thymosin alpha-1 enhances invariant NKT-cell cytotoxicity and has shown benefit in hepatocellular carcinoma and colorectal cancer, illustrating how immune adjuvants can be integrated with TCM-based strategies within the same gut–liver framework [20, 92, 95]. These observations support the view that interventions acting on Axis III and Axis IV can help restore local immune surveillance in organs where metabolism, microbiota, and immune tone are tightly coupled.

### Integrated view

Across immune lineages, the same metabolic levers recur: glycolytic stress and lactate accumulation, mitochondrial integrity and ICD/ferroptosis, lipid–bile-acid circuits in myeloid niches, and intrinsic DC/T-cell bioenergetics. TCM-derived prescriptions and monomers engage these levers in a coordinated fashion—supporting DC cross-priming, sustaining CD8⁺ and Th1 T-cell function, repolarizing TAMs, constraining MDSCs, and restoring NKT-cell surveillance—rather than acting on isolated markers or pathways.

This integrated perspective helps reinterpret classical notions such as *Fuzheng Quxie* (“supporting the vital Qi and dispelling pathogenic factors”), *Tiaogan Hepi* (“harmonizing Liver–Spleen”), and *Peiben Chuzhuo* (“rooting and eliminating turbid-evil”) therapy in metabolic terms. By simultaneously modulating tumor glycolysis, ferroptosis/ICD, lipid–bile-acid metabolism, and immune cell bioenergetics, TCM interventions can shift the balance between tolerance and immunity in the TME and provide a mechanistic rationale for their use as immunometabolic co-therapies in modern oncology.

## Integration of TCM theory and modern immunometabolism

TCM does not describe disease in terms of “glycolysis,” “ferroptosis,” or “DC/T-cell metabolic fitness.” Instead, it uses doctrines such as *Fuzheng Quxie* (“supporting the vital Qi and dispelling pathogenic factors”), *Tiaogan Hepi* (“harmonizing Liver–Spleen”), *Peiben Chuzhuo* (“rooting and eliminating turbid-evil”), and *Bianzheng Lunzhi* (“syndrome differentiation and treatment”) to capture recurrent patterns of organ dysfunction, pathogenic burden, and constitutional weakness. When reinterpreted through an immunometabolic lens, these doctrines map onto combinations of the four axes introduced above: glycolysis and lactate (Axis I), mitochondrial integrity and ferroptosis/ ICD (Axis II), lipid–bile-acid–centered myeloid niches (Axis III), and DC/T-cell metabolic fitness (Axis IV) [1, 11, 23, 25, 102].

This doctrine-to-axis mapping should not be interpreted as a direct one-to-one equivalence between classical TCM concepts and individual molecular pathways. Instead, it is proposed as a translational vocabulary that links pattern-based clinical reasoning to measurable immunometabolic phenotypes. In this sense, doctrines such as Fuzheng Quxie, Tiaogan Hepi, Peiben Chuzhuo, and Bianzheng Lunzhi are used not as substitutes for molecular mechanisms, but as higher-level clinical descriptors that may guide hypothesis generation, biomarker selection, and combination design. The validity of this mapping will require further testing through standardized formulations, axis-matched biomarkers, and prospective clinical studies.

**Fig. 3:**
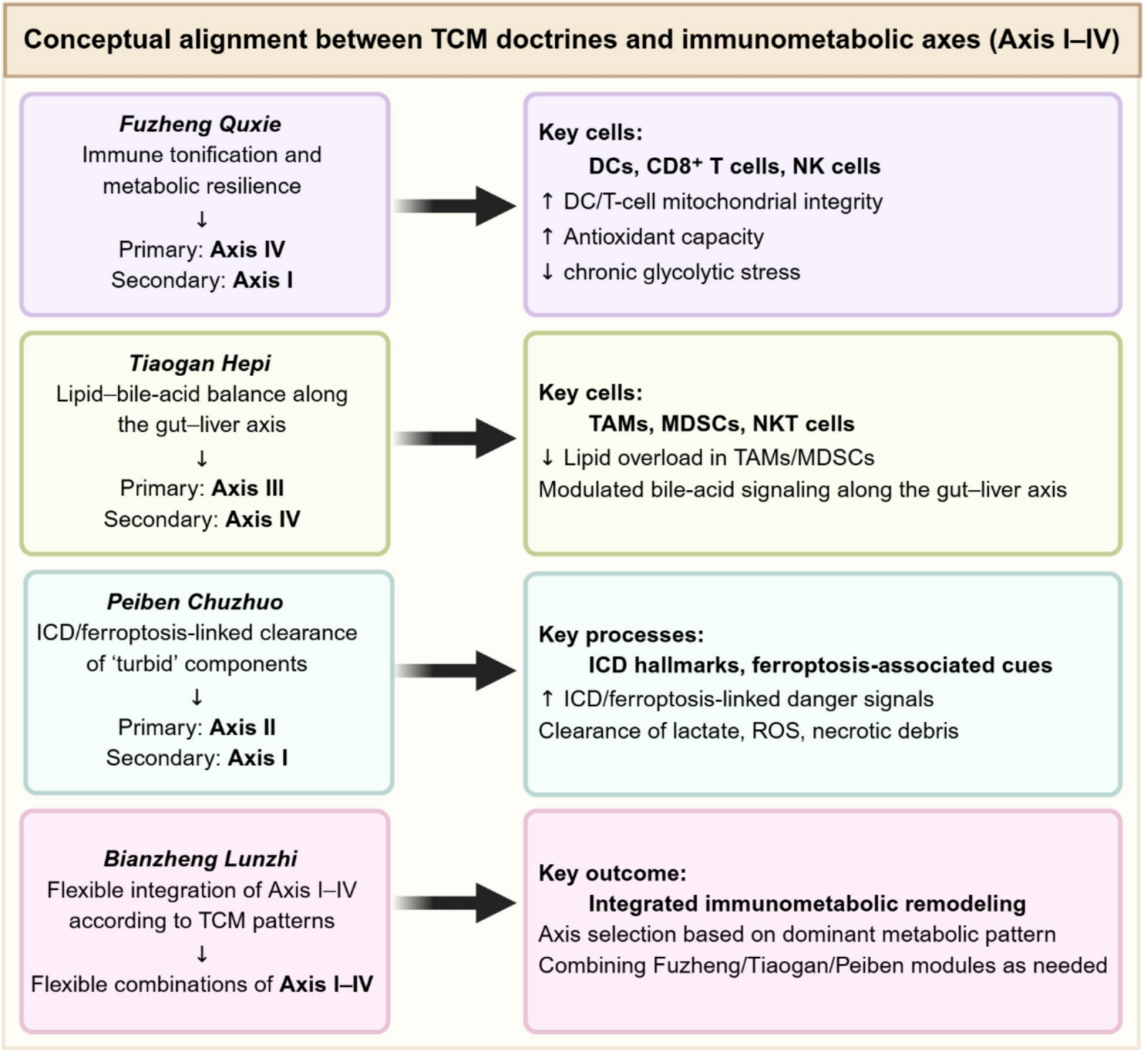
Translational mapping between TCM doctrines and immunometabolic axes. Classical TCM doctrines are mapped to the four immunometabolic axes as a translational framework rather than as direct molecular equivalences. Fuzheng Quxie is mainly aligned with DC/T-cell metabolic fitness and partial relief of glycolysis–lactate stress; Tiaogan Hepi is mainly linked to lipid–bile-acid–centered myeloid niches and gut–liver immunometabolism; Peiben Chuzhuo is mainly associated with mitochondrial stress, ferroptosis, and ICD; and Bianzheng Lunzhi provides an adaptive principle for phenotype-guided, axis-based co-therapy. This mapping is intended to guide hypothesis generation, biomarker selection, and future trial design

Rather than enforcing a rigid one-to-one correspondence, each doctrine can be viewed as a bias toward certain axes in a given clinical context. *Fuzheng Quxie* tilts toward Axis IV (host defense) with a secondary influence on Axis I; *Tiaogan Hepi* primarily involves Axis III with an Axis IV component; *Peiben Chuzhuo* emphasizes Axis II with links to Axis I; and *Bianzheng Lunzhi* provides an overarching framework for selecting and combining axis-specific strategies. In contemporary terms, these doctrines can be understood as early systems-level attempts to integrate metabolic, inflammatory, and constitutional information—concepts that now resonate with systems oncology and biomarker-driven therapy [1, 48, 76]. A pragmatic mapping between doctrines, metabolic axes, and representative formulas/compounds is summarized in Fig. 3 and Table 2.

**Table 2:** Axis-based TCM modules for phenotype-guided co-therapy

Metabolic phenotype	Key biomarkers	Priority axes	Example TCM	Combination partners
Glycolysis-dominant, lactate-rich, immune-excluded tumors	High LDH; glycolytic signatures; low TIL; FDG-PET–avid	Axis I + Axis IV	GQD; Astragalus regimens; Quxie Capsule	ICIs; chemotherapy; RT; DC/T-cell vaccines
Immune-cold, low ICD/ferroptosis, weak TIL infiltration	Low ICD markers; poor DC activation	Axis II + Axis IV	Rg3 nanomedicines; celastrol/gambogic-acid nanoplatforms; Fe₃O₄/COF hydrogels	PD-1/PD-L1 & CTLA-4 blockade; neoantigen vaccines; RT/phototherapy
Lipid–bile-acid–driven myeloid-dense gut–liver tumors (HCC/CRC-liver involvement)	Abnormal bile acids; steatohepatitis; high TAM/MDSC	Axis III ± Axis IV	Jianpi Jiedu; Jianpi Huayu; Compound Kushen Injection	Systemic therapy; ICIs; anti-angiogenic agents; NKT-based adjuvants
Systemically frail/treatment-fatigued patients	Low PS; lymphopenia; infection-prone; poor chemo tolerance	Axis IV ± Axis I	Astragalus regimens; Hochu-ekki-to; Huaier; Ganoderma; SQYC	Chemotherapy; targeted therapy; ICIs with monitoring
Heterogeneous tumors with mixed metabolic niches	Spatially variable glycolysis/lipid signatures; mixed immune profiles	Adaptive Axis I–IV	Bianzheng-guided combinations	Biomarker-driven adaptive regimens

*LDH* lactate dehydrogenase, *TIL* tumor-infiltrating lymphocyte, *FDG-PET *^1^⁸F-fluorodeoxyglucose positron emission tomography, *HCC* hepatocellular carcinoma, *CRC* colorectal cancer, *TAM* tumor-associated macrophage, *MDSC* myeloid-derived suppressor cell, *PS* performance status, *RT* radiotherapy, *ICI* immune checkpoint inhibitor, *NKT* natural killer T cell

### *Fuzheng Quxie* (“supporting the vital Qi and dispelling pathogenic factors”)—axis IV with axis I

*Fuzheng Quxie* emphasizes strengthening host defenses while reducing pathogenic burden. Mechanistically, this doctrine aligns most closely with Axis IV (DC/T-cell metabolic fitness), with a complementary effect on Axis I (relieving glycolytic and lactate stress). In metabolic terms, *Fuzheng Quxie*–oriented regimens support immune cell bioenergetics, enhance antigen presentation and T-cell function, and lightly depress excessive tumor glycolysis in glycolysis-high, lactate-rich microenvironments [48, 81, 95, 102].

Astragalus-based formulations are prototypical examples. Astragalus polysaccharide and Astragalus-containing decoctions improve DC maturation and T-cell activation, increase Th1-polarized cytokine profiles, and enhance responses to chemotherapy or checkpoint inhibitors in several malignancies [25, 79]. At the same time, these regimens can modestly downregulate glycolytic enzymes, lower lactate accumulation, and improve antioxidant capacity, thereby easing metabolic pressure on immune cells without globally suppressing glycolysis.

Formulas such as SQYC, as well as Hochu-ekki-to and similar tonifying prescriptions, extend this pattern. They are frequently used in debilitated or treatment-exhausted patients, where they improve performance status, support hematopoiesis, and stabilize lymphocyte counts. Preclinical and clinical data suggest that these effects are accompanied by improved DC and T-cell function and better maintenance of immune repertoires during chemoimmunotherapy, especially in glycolysis-high, immune-excluded tumors [7, 81]. From an axis perspective, *Fuzheng Quxie* corresponds to supporting Axis IV while gently modulating Axis I.

### *Tiaogan Hepi* (“harmonizing liver–spleen”)—axis III with axis IV

*Tiaogan Hepi* highlights the functional interaction between the Liver and Spleen systems in TCM, covering emotional regulation, digestive function, bile and lipid handling, and the gut–liver axis. In immunometabolic terms, this doctrine primarily points to Axis III, centered on lipid and bile-acid reprogramming of myeloid niches, with a secondary influence on immune cell metabolic competence (Axis IV) [11, 12, 57]. It is particularly relevant in hepatocellular carcinoma (HCC), colitis-associated cancer, and other malignancies where chronic inflammation, dyslipidemia, and bile-acid disruption drive tumor evolution.

Jianpi Jiedu and Jianpi Huayu decoctions—commonly prescribed to “fortify the Spleen and detoxify the Liver”—illustrate this alignment. In preclinical models, they improve intestinal barrier function, remodel microbiota composition, normalize bile-acid pools, and reduce inflammatory signaling along the gut–liver axis. These changes are accompanied by repolarization of TAMs, reduced MDSC burden, and improved DC and T-cell function, indicating that *Tiaogan Hepi*–type prescriptions primarily reprogram lipid–bile-acid–dominated myeloid niches (Axis III) while secondarily supporting Axis IV [60, 76].

Additional support for this mapping comes from studies on hepatocarcinogenesis and liver fibrosis. *Tiaogan Hepi*–oriented formulas and injections, including Compound Kushen Injection and related regimens, ameliorate fibrotic remodeling, improve hepatic metabolism, and modulate Kupffer cell and other liver-resident myeloid subsets. Quxie Capsule and similar approaches, by reshaping microbiota and histone demethylase activity (e.g. KDM4D), further highlight the capacity of *Tiaogan Hepi* strategies to influence gut–liver immunometabolism in tumors whose biology is tightly coupled to the hepatic axis [59, 92].

### *Peiben Chuzhuo* (“rooting and eliminating turbid-evil”)—axis II with axis I

*Peiben Chuzhuo* refers to “treating the root” while eliminating “turbid-evil”—a term that encompasses pathological accumulations such as phlegm, blood stasis, toxins, or rapidly proliferating masses. When reframed in immunometabolic terms, this doctrine maps onto Axis II, prioritizing mitochondrial integrity and ferroptosis/ICD, with a secondary link to Axis I (modulating glycolytic stress). It emphasizes not just killing tumor cells, but doing so in a way that produces robust danger signals and supports immune activation [16, 34, 64].

Modern formulations provide concrete mechanistic expressions of *Peiben Chuzhuo*. Xihuang Pill, Kejinyan decoction, and related “anti-mass” prescriptions, often used in patients with advanced or metastatic disease, have been shown to inhibit tumor growth, reduce metastatic spread, and alter angiogenic and inflammatory signatures. Experimental work suggests that they can induce or amplify ICD-like death, increase DAMP release, and sensitize tumors to chemotherapy or radiotherapy, thereby linking traditional “phlegm- and stasis-resolving” concepts to ICD- and ferroptosis-oriented oncology [17, 22, 64].

TCM-inspired nanomedicines and in situ delivery systems further operationalize *Peiben Chuzhuo* by confining oxidative and ferroptotic stress to tumor tissues and post-surgical beds. Platforms based on Schottky heterojunction photocatalysts, Fe₃O₄ nanohybrids, covalent organic frameworks, or injectable hydrogels can trigger iron-dependent lipid peroxidation or ROS bursts in tumor cells while simultaneously carrying immunomodulators that stimulate DCs and T cells [17, 18, 35]. Many of these systems also incorporate tumor glycolysis modulators, highlighting the functional intersection of Axis II (ICD/ferroptosis) with Axis I in their design [67, 81, 84, 103].

Within this framework, *Peiben Chuzhuo* corresponds to selective activation of Axis II, often combined with partial Axis I modulation, to ensure that tumor cell death is both effective and immunologically productive, rather than metabolically disorganized or immunologically silent.

### *Bianzheng Lunzhi* (“syndrome differentiation and treatment”)—phenotype-guided, axis-based co-therapy

*Bianzheng lunzhi* emphasizes tailoring treatment to a patient’s overall pattern, integrating symptoms, signs, tongue and pulse findings, and other contextual information. In an immunometabolic reinterpretation, it can be viewed as a strategy for selecting and combining Axis I–IV interventions based on a tumor’s metabolic and immune phenotype, and for revising them as the disease evolves [1, 25, 57].

In practice, tumors can be grouped into several broad immunometabolic phenotypes that loosely correspond to TCM patterns. Glycolysis-dominant, lactate-rich, immune-excluded tumors align with Axis I–heavy states; lipid–bile-acid–dominated, myeloid-rich niches correspond more closely to Axis III; lesions marked by extensive ferroptosis/ICD resistance or mitochondrial dysfunction resonate with Axis II; and hosts with profound immune debility or treatment-induced exhaustion highlight the need for Axis IV support. *Bianzheng lunzhi*, reinterpreted in this way, becomes a framework for combining *Fuzheng Quxie*, *Tiaogan Hepi*, and *Peiben Chuzhuo*–type interventions with modern therapies, mixing Axis I–III modulators with Axis IV–oriented “supportive” modules to sustain T-cell responses [16, 17, 34, 64, 82, 84, 103].

Initial stratification could rely on accessible biomarkers—serum LDH, lipid and bile-acid panels, markers of ferroptosis sensitivity, immune cell metabolic signatures, imaging-based hypoxia or perfusion indices—together with clinical and TCM pattern assessments. Over time, longitudinal profiling of tumor metabolism, immune cell states, and systemic parameters could guide dynamic adjustment of formulas, doses, and combinations, mirroring the TCM practice of modifying prescriptions as the pattern changes. To make this doctrine–axis mapping more operational, we propose a phenotype-to-axis workflow that remains compatible with biomarker-driven oncology. First, integrate TCM pattern features with measurable immunometabolic surrogates to define the dominant phenotype. Second, assign a primary axis (the dominant constraint on antigen presentation or effector function) and one or more secondary coupled axes, reflecting predictable crosstalk (e.g., lactate–redox linking Axis I ↔ II; lipid peroxidation/ferroptosis linking Axis III ↔ II; nutrient competition linking Axis I ↔ IV). Third, select doctrine-aligned TCM modules—Fuzheng Quxie (Axis IV with Axis I), Tiaogan Hepi (Axis III with Axis IV), and Peiben Chuzhuo (Axis II with Axis I)—and specify axis-matched pharmacodynamic markers together with immune-functional endpoints. Finally, implement axis-based co-therapy alongside standard treatments (e.g., chemotherapy or immune checkpoint blockade) and iteratively adjust formulas/doses/combinations according to longitudinal biomarker dynamics and immune function, translating traditional pattern adjustment into a testable, mechanism-informed strategy for future clinical evaluation.

In summary, aligning TCM doctrines with immunometabolic axes does not reduce traditional theory to isolated pathways; rather, it provides a structured vocabulary to translate pattern-based reasoning into mechanism-informed co-therapy. Within this framework, Fuzheng Quxie primarily supports immune metabolic fitness (Axis IV) while secondarily easing glycolytic/lactate stress (Axis I); Tiaogan Hepi primarily targets lipid–bile-acid–shaped myeloid niches (Axis III) with secondary benefits for immune competence (Axis IV); and Peiben Chuzhuo emphasizes mitochondrial/redox stress and immunogenic tumor death (Axis II) with links to Axis I. Bianzheng Lunzhi serves as an adaptive integration principle that selects and combines these modules based on evolving immunometabolic phenotypes. The proposed phenotype-to-axis decision path makes this correspondence actionable by linking doctrine-informed module choice to axis-matched biomarkers, pharmacodynamic readouts, and immune-functional endpoints, thereby supporting rational design of TCM-informed combination therapies for future clinical testing.

Therefore, the proposed TCM doctrine–axis framework should be understood as a bridge between clinical pattern recognition and experimentally measurable biology. Its practical value will depend on whether specific TCM patterns and formula modules can be prospectively linked to reproducible metabolic features, immune-cell states, pharmacodynamic markers, and therapeutic outcomes. This requirement also defines an important direction for future trials: to test whether syndrome-informed selection of TCM-based co-therapies improves outcomes beyond non-stratified empirical use.

## Challenges, knowledge gaps, and future perspectives

Despite increasingly rich preclinical data, translation of TCM-based immunometabolic strategies into routine oncology practice remains challenging. Key obstacles include methodological limitations of current evidence, the complexity and safety implications of polypharmacology, incomplete mechanistic and biomarker frameworks, and the scarcity of rigorously designed, axis-based clinical trials. Addressing these issues is crucial if TCM-derived interventions are to be integrated into modern, mechanism-driven cancer therapy.

### Methodological limitations of current evidence

Most available studies rely on reductionist systems—tumor cell lines maintained under simplified culture conditions, xenografts in immunodeficient mice, or small clinical cohorts without stratification by metabolic phenotype [11, 44, 57]. In vitro experiments frequently use supraphysiological concentrations of herbal extracts or monomers, making it difficult to judge whether the reported effects can be reproduced at clinically feasible doses.

To make the strength and limitations of the current evidence more explicit, Table 3 provides a concise evidence map of representative interventions. The evidence map also highlights several recurring weaknesses. First, many studies remain confined to in vitro experiments or animal models and do not include clinically relevant immune-metabolic endpoints. Second, even when clinical data are available, most studies evaluate conventional outcomes such as tumor response, survival, symptom improvement, or treatment tolerance, whereas lactate dynamics, lipid/bile-acid profiles, ICD/ferroptosis markers, DC function, T-cell metabolic fitness, and myeloid phenotypes are rarely prespecified. Third, formula composition, extraction procedures, dosing strategies, and treatment duration are often insufficiently standardized, limiting reproducibility across studies. These limitations support the need for standardized formulations, axis-matched biomarkers, and prospective biomarker-stratified trials.

**Table 3:** Evidence map of representative TCM-derived immunometabolic interventions

Representative intervention	Primary axis	Evidence level	Key evidence	Key limitation
Gegen Qinlian decoction	Axis I	In vitro + animal	PKM2/glycolysis; lactate; myeloid/T-cell infiltration	Limited clinical immune-metabolic endpoints
Astragalus polysaccharide/Astragalus-based formulas	Axis I/IV	In vitro + animal + clinical supportive evidence	Glycolysis; DC maturation; CD8⁺ T-cell responses	Formula heterogeneity; limited stratified trials
Ginsenoside Rg3 and related ginsenosides	Axis II/IV	In vitro + animal + clinical supportive evidence	PD-L1; ferroptosis/apoptosis; CD8⁺ T-cell activation	Variable immune-metabolic endpoints
Dihydroartemisinin	Axis II	In vitro + bioinformatics	Ferroptosis-related genes; survival-related pathways	Limited in vivo immune-functional validation
Compound Kushen Injection	Axis III	Clinical use + animal/mechanistic data	Lipid metabolism; hepatic inflammation; myeloid suppression	Need prospective immune-metabolic endpoints
Jianpi Jiedu/Jianpi Huayu decoctions	Axis III / IV	Animal + omics	Bile-acid/lipid metabolism; microbiota; TAM/MDSC remodeling	Limited standardization and prospective validation
Huaier	Axis IV	Clinical supportive + preclinical	DC maturation; IFN-γ⁺ T cells; CD8⁺ T-cell infiltration	Incomplete metabolic readouts
TCM-inspired nanoplatforms/hydrogels/microneedles	Axis II/ V	Animal proof-of-concept	ROS; ferroptosis/ICD; cGAS–STING; DC priming	Safety and manufacturing concerns

*cGAS–STING* cyclic GMP–AMP synthase–stimulator of interferon genes, *DC* dendritic cell, *ICD* immunogenic cell death, *IFN-γ* interferon-gamma, *MDSC* myeloid-derived suppressor cell, *PD-L1* programmed death-ligand 1, *PKM2* pyruvate kinase M2, *ROS* reactive oxygen species, *TAM* tumor-associated macrophage, *TCM* Traditional Chinese Medicine, *TME* tumor microenvironment

Many reports also focus on a narrow set of endpoints—tumor volume, a limited cytokine panel, or a few signaling proteins—without systematically characterizing how TCM interventions reshape metabolic architecture and immune-cell composition within the TME. Only a minority of studies integrate multi-omic profiling, metabolomics, spatial mapping, and detailed immune phenotyping. As a result, it often remains unclear whether a given prescription truly modulates immunometabolism or acts mainly through altered pharmacokinetics, improved tolerability, or broader anti-inflammatory effects [1, 39].

Although preparations such as Compound Kushen Injection and spleen-fortifying formulas including Jianpi Jiedu and Jianpi Huayu decoctions have entered clinical use in some settings, most clinical trials remain small, heterogeneous, and shallow in mechanistic profiling [25, 42, 48, 57]. Future studies should include adequately powered randomized designs with appropriate control arms, standardized and chemotype-defined formulations, prespecified immunometabolic readouts, and long-term follow-up to clarify which combinations provide genuine benefit and in which patient subgroups.

Therefore, the current evidence should be interpreted as suggestive rather than definitive. The four-axis framework provides a useful structure for organizing mechanistic findings, but it does not yet establish clinical efficacy or determine patient selection on its own. Future validation will require reproducible pharmacodynamic readouts, harmonized immune-metabolic endpoints, and prospective studies that directly test whether axis-matched TCM interventions improve therapeutic outcomes.

### Complexity, polypharmacology, safety, and herb–drug interactions

The multicomponent, multitarget nature of TCM underpins its capacity to act across multiple axes, but also complicates mechanistic interpretation and raises safety concerns. In patients already receiving immune checkpoint inhibitors, targeted therapies, or multidrug chemotherapy, additional herbal preparations may influence overlapping pathways and drug-handling systems in unpredictable ways [25, 48, 76, 78].

Safety assessment is particularly important when TCM-derived agents are combined with chemotherapy, radiotherapy, targeted therapy, endocrine therapy, anticoagulants, corticosteroids, or immune checkpoint inhibitors. Multicomponent herbal preparations may alter drug absorption, hepatic metabolism, renal clearance, or membrane transporter activity, including pathways related to cytochrome P450 enzymes, UDP-glucuronosyltransferases, organic anion/cation transporters, and P-glycoprotein. These interactions may increase or decrease exposure to cytotoxic drugs, tyrosine kinase inhibitors, immune-modulating agents, or supportive medications, thereby influencing both efficacy and toxicity.

In the context of immunotherapy, additional caution is required because immunomodulatory herbal agents may theoretically influence the balance between antitumor immunity and immune-related adverse events. Preparations that enhance T-cell activation, cytokine production, antigen presentation, or myeloid reprogramming may be beneficial in immune-excluded tumors, but they could also modify inflammatory toxicity in susceptible patients. Therefore, future clinical studies should include systematic pharmacovigilance, standardized adverse-event grading, liver and renal function monitoring, hematologic toxicity assessment, and, where possible, pharmacokinetic–pharmacodynamic evaluation. For modern TCM-inspired nanomedicines, further attention should be paid to biodistribution, long-term retention, carrier immunogenicity, off-target tissue accumulation, and manufacturing reproducibility.

In the era of immunotherapy, where subtle shifts in immune tone can determine whether a patient experiences durable tumor control or severe immune-related toxicity, these issues require particular attention. Some TCM components may potentiate antitumor immunity, whereas others may dampen immune responses, bias them toward tolerance, or alter drug metabolism, with context-dependent net effects in heavily pretreated patients [81, 87, 95].

To manage this complexity, systematic pharmacokinetic–pharmacodynamic studies are needed, together with careful monitoring of adverse events and consistent use of validated toxicity grading systems. Regulatory frameworks must also evolve to accommodate TCM-based co-therapies—enforcing quality control, batch consistency, and transparent reporting—while still allowing pattern-guided individualization in appropriate settings [62, 104].

### Mechanistic and biomarker gaps

Although this review has organized TCM interventions along four immunometabolic axes, the underlying mechanisms remain incompletely defined. Many studies link formulas or monomers to broad signaling cascades—such as PI3K–Akt–mTOR, STAT3, HIF-1α, or NF-κB—without clearly explaining how these changes give rise to specific immunometabolic states (for example, glycolysis-dominant vs lipid-driven vs ferroptosis-prone niches) [17, 64].

Time-resolved and spatially resolved analyses are also limited. Few investigations track how TCM interventions remodel metabolic and immune features over successive treatment cycles, or how they differentially affect tumor cores, invasive fronts, and draining lymph nodes [25, 73]. Candidate biomarkers that might guide axis-based TCM modules—such as lactate-related signatures for Axis I, bile-acid and myeloid markers for Axis III, or ICD/ferroptosis footprints for Axis II—are still largely exploratory. Defining, validating, and harmonizing such biomarkers will be essential for translating the four-axis framework into practical clinical stratification.

### Designing biomarker-stratified clinical trials: from empirical use to mechanism-based practice

Future clinical trials should move beyond simply adding “TCM” to standard regimens and instead test explicit mechanistic hypotheses. Individual protocols should specify whether the selected Axis I–IV module is intended primarily to lower lactate burden, reprogram myeloid niches, enhance ICD/ferroptosis, or stabilize DC/T-cell metabolic fitness in a clearly defined immunometabolic phenotype [1, 57].

Several design principles follow from this perspective. First, axis-specific endpoints should be embedded alongside conventional clinical outcomes. For Axis I, this may include imaging or transcriptomic measures of glycolysis and lactate dynamics; for Axis II, biomarkers of ICD/ferroptosis and DC cross-priming; for Axis III, bile-acid profiling, lipidomics, and detailed myeloid phenotyping; and for Axis IV, high-dimensional characterization of DC/T-cell subsets, exhaustion markers, and metabolic fitness [1, 3].

Second, phenotype-guided stratification should be implemented from the outset. *Bianzheng Lunzhi* can be operationalized as metabolic patterning—glycolysis-dominant, lipid–bile-acid–driven, ferroptosis-deficient, or immune-cold tumors—with rational allocation of Axis I–IV modules to each category. Adaptive trial designs could then refine module combinations and dosing based on interim biomarker responses and evolving metabolic signatures [25, 42].

Ultimately, axis-based trials that integrate robust biomarkers, advanced delivery systems, and rigorous safety monitoring will be required to shift TCM-derived immunometabolic interventions from empirical adjuncts to well-defined, mechanism-based co-therapies in contemporary oncology.

In addition to tissue-based and circulating biomarkers, molecular imaging may help refine patient selection and pharmacodynamic monitoring in future axis-based trials. For example, antibody-based PD-L1 imaging using ⁸⁹Zr-labeled probes has been explored as a non-invasive strategy to visualize immune-checkpoint-related tumor features in lung cancer models. Although this work is not specific to TCM, it illustrates how imaging biomarkers could be integrated into trials evaluating TCM-derived co-therapies combined with immune checkpoint blockade or other immunotherapies [105].

A practical translational path should include three steps. First, the intervention itself must be standardized through botanical authentication, chemical fingerprinting, batch-to-batch consistency testing, dose definition, and quality-control markers. Second, patient selection should be guided by axis-matched biomarkers, such as glycolytic/lactate signatures for Axis I, ICD/ferroptosis markers for Axis II, lipid–bile-acid and myeloid signatures for Axis III, and DC/T-cell metabolic fitness indicators for Axis IV. Third, clinical trials should incorporate prespecified immune-metabolic endpoints in addition to conventional outcomes such as objective response rate, progression-free survival, overall survival, quality of life, symptom burden, and treatment-related toxicity. Such a design would allow TCM-derived interventions to be tested as mechanism-defined co-therapies rather than empirically added supportive treatments.

### Emerging immunotherapies and data-driven translation

Beyond immune checkpoint blockade, emerging modalities such as CAR-T cell therapy and therapeutic cancer vaccination provide additional contexts in which TCM-informed immunometabolic remodeling may be explored. Solid tumors remain challenging for CAR-T due to antigen heterogeneity, poor trafficking, hypoxia/lactate stress, and exhaustion programs within the TME. Mechanistically, these barriers map naturally onto Axis I (glycolysis/lactate and nutrient competition) and Axis IV (immune-cell metabolic fitness), with secondary coupling to Axis II (mitochondrial/redox stress). Early evidence supports the hypothesis that selected herbal formulations or derived modules may be evaluated as adjunct strategies to mitigate microenvironmental constraints and improve CAR-T infiltration, persistence, and effector function; however, rigorous validation using prespecified pharmacodynamic markers and immune-functional endpoints will be required before clinical translation [106, 107].

Therapeutic cancer vaccines—particularly in situ vaccination strategies—also align well with the four-axis framework, because vaccine efficacy ultimately depends on efficient antigen release, danger signaling, dendritic-cell activation/cross-priming, and durable T-cell responses. A growing body of work illustrates how bioactive natural products and TCM-inspired nanoplatforms can induce immunogenic tumor death, amplify ICD/ferroptosis-associated cues (Axis II), and promote DC priming, often synergizing with checkpoint blockade [70, 108, 109]. In addition, certain TCM techniques and formula-derived interventions may modulate systemic neuro-immune or stress pathways that shape vaccine responsiveness and myeloid tone, offering distinctive translational angles that warrant careful mechanistic dissection and standardized evaluation [110].

Finally, artificial intelligence and systems-level analytics can accelerate the operationalization of axis-based TCM co-therapy. AI-assisted network pharmacology, together with multi-omics integration (single-cell/spatial transcriptomics, metabolomics/lipidomics, and immune profiling), can prioritize active component–target modules, identify coupling nodes across Axis I–IV, and support patient stratification into immunometabolic phenotypes for adaptive trial designs [111, 112]. Embedding such approaches into prospective studies may enable an iterative workflow—biomarker-defined axis assignment, module selection, pharmacodynamic monitoring, and longitudinal adjustment—thereby translating pattern-guided practice into reproducible, mechanism-informed strategies, while recognizing that data-driven inferences remain hypothesis-generating until prospectively validated.

## Conclusions

### Summary of key concepts

Cancer progression is governed not only by oncogenic mutations but also by a profoundly altered metabolic ecosystem in which tumor cells, stromal elements, and immune populations compete for scarce nutrients and adapt to chronic stress. High glycolytic flux, lactate accumulation, dysregulated lipid–bile-acid networks, disturbed amino-acid utilization, and redox imbalance together create microenvironments that favor immune escape, therapy resistance, and metastatic spread [1, 3, 11]. Even when tumors express abundant antigens and druggable immune checkpoints, DCs and T cells often fail to mount durable responses if their bioenergetic state is not simultaneously restored [4, 10].

Within this setting, this review has organized TCM-derived interventions into four recurrent immunometabolic axes: (i) glycolysis and lactate (Axis I); (ii) mitochondrial integrity and ICD/ferroptosis (Axis II); (iii) lipid–bile-acid–centered myeloid niches (Axis III); and (iv) DC/T-cell metabolic fitness (Axis IV). Illustrative examples include classical prescriptions such as GQD, Jianpi Jiedu and Jianpi Huayu formulas, Astragalus-based regimens, Compound Kushen Injection, SQYC, and Hochu-ekki-to, together with isolated monomers and modern delivery systems that integrate herbal components with contemporary materials science [34, 35, 103].

**Fig. 4:**
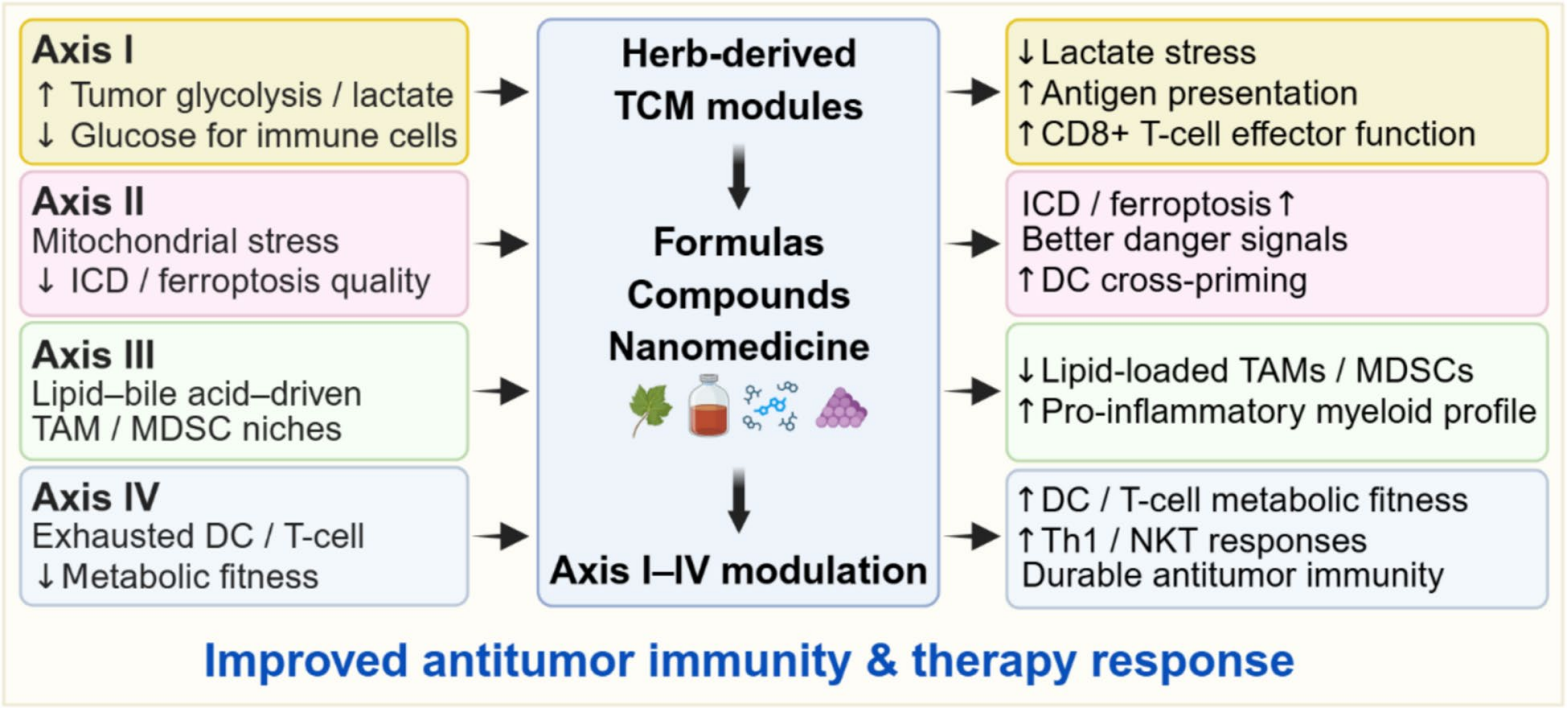
Axis-based framework linking herb-derived TCM modules to immunometabolic remodeling. Herb-derived TCM modules—delivered as formulas, isolated compounds, or nanomedicines—can be positioned along four dominant immunometabolic axes. Axis I reflects glycolysis- and lactate-driven stress limiting nutrient availability and antigen presentation; Axis II captures mitochondrial/redox stress shaping ICD and ferroptosis quality; Axis III denotes lipid–bile-acid–driven TAM/MDSC niches; and Axis IV represents exhausted DC/T-cell states with reduced metabolic fitness. Overlap between axes is expected and arises through coupling nodes (e.g., lactate–redox signaling, lipid peroxidation/ferroptosis interfaces, nutrient competition, and bile-acid/AhR signaling). Thus, a given intervention may engage a primary axis with secondary coupled axes, together providing a conceptual basis for improving antitumor immunity and potentially enhancing therapeutic responses

Taken together, these patterns support a view of TCM not merely as symptomatic “supportive care” but as a potential source of multi-target immunometabolic co-therapies. By modestly restraining tumor glycolysis, promoting ICD/ferroptosis in selected contexts, remodeling lipid–bile-acid–driven myeloid environments, and stabilizing DC/T-cell bioenergetics, TCM-based modules may deepen responses to checkpoint inhibitors, therapeutic vaccines, adoptive cell therapies, and chemotherapy, while in some cases also mitigating treatment-related toxicity [62, 64] (Fig. 4).

### Outlook: positioning TCM in future immunometabolic oncology

Several priorities emerge for future research and clinical translation. First, more chemotype-resolved, mechanism-driven studies are needed to identify which compounds and formulas truly act on specific immunometabolic axes and through which molecular pathways. Multi-omic, spatially resolved, and time-resolved analyses should increasingly replace generic “multi-target” descriptions, enabling clearer attribution of Axis I–IV effects to defined chemotypes and prescriptions [1, 42, 64].

Second, operationalizing *Bianzheng Lunzhi* in oncology will require closer connections between clinical pattern differentiation and metabolic biomarkers. Composite signatures that combine lactate and glycolytic indicators (Axis I), ICD/ferroptosis footprints (Axis II), bile-acid and myeloid profiles (Axis III), and DC/T-cell exhaustion and fitness indices (Axis IV) will be necessary to guide the selection and dynamic adjustment of TCM modules in individual patients [75].

Third, axis-based clinical trial designs and delivery innovations are essential. Prospective studies should explicitly test axis-focused hypotheses, integrating TCM modules that are rationally chosen for specific metabolic phenotypes and administered through optimized platforms such as nanocarriers, hydrogels, and microneedles, with embedded pharmacokinetic and pharmacodynamic readouts [33, 81].

Finally, issues of safety, herb–drug interactions, and regulatory oversight must be addressed as TCM co-therapies are combined with checkpoint inhibitors, CAR-T cells, and other complex multi-agent regimens. Particular attention should be paid to interactions between herbal products and conventional drugs, the risk of immune-related adverse events, and the safe implementation of TCM-based immunometabolic modules outside highly specialized centers [67, 71, 87, 103]. If these challenges can be met, TCM may ultimately occupy a clearly defined niche within immunometabolic oncology—as a family of mechanistically grounded, biomarker-guided co-therapies capable of tackling some of the most difficult problems in current cancer care.

At the current stage, however, most TCM-derived immunometabolic interventions should be viewed as promising but incompletely validated co-therapeutic candidates. Their successful translation will depend on moving from descriptive pharmacology toward standardized products, axis-matched biomarkers, reproducible immune-metabolic endpoints, rigorous safety assessment, and prospective clinical trials that can determine which patients are most likely to benefit.

## List of key compounds and formulas

### Single compounds and herb-derived extracts

*Astragalus polysaccharide*—purified polysaccharide fraction from *Astragalus membranaceus* that modulates PI3K–AKT–mTOR/HIF-1α signaling, attenuates tumor glycolysis, and enhances DC/T-cell responses.

*Berberine*—isoquinoline alkaloid that reduces glycolytic flux and lactate burden while supporting immune effector functions.

*Celastrol*—quinone methide triterpenoid that induces mitochondrial stress, ROS generation, ICD/ferroptosis, and enhances antitumor T-cell responses, especially in nanoplatforms.

*Emodin*—anthraquinone that reduces macrophage lipid overload, remodels hepatic myeloid niches, and contributes to TAM repolarization.

*Gambogic acid*—garcinia-derived compound used in nanoplatforms to trigger mitochondrial damage, ROS bursts, and ICD, thereby improving checkpoint blockade.

*Ginsenoside Rg3 and related ginsenosides*—triterpenoid saponins that induce tumor cell apoptosis/ferroptosis, lower PD-L1 expression, and enhance CD8⁺ T-cell proliferation and memory formation.

*Licochalcone A*—flavonoid that attenuates glycolytic flux and lactic acid production in tumor cells while preserving antigen presentation.

*Scutellaria barbata extracts*—flavonoid- and diterpene-rich extracts that contribute to mitochondrial stress, ICD induction, and immunomodulation.

*Stigmasterol*—plant sterol that improves lipid handling, dampens inflammatory signaling, and promotes favorable TAM phenotypes.

*Ursolic acid*—pentacyclic triterpenoid that can trigger ICD/ferroptosis and modulate myeloid metabolism while sparing immune cells.

### Classical formulas and patent medicines

*Astragalus-based prescriptions*—Qi-tonifying decoctions and pills centered on *Astragalus membranaceus* that reduce tumor glycolysis, alleviate lactate stress, and enhance DC/T-cell bioenergetics.

*Compound Fuling Granule*—multi-herb formula that modulates lipid–bile-acid metabolism, TAM/MDSC phenotypes, and gut–liver immunometabolism.

*Compound Kushen Injection*—alkaloid-enriched injectable preparation that rebalances hepatic lipid–inflammation profiles and reshapes hepatic myeloid niches, particularly in HCC.

*Gegen Qinlian decoction (GQD)*—multi-herb prescription that downregulates PKM2, reduces lactate accumulation, and improves myeloid/T-cell trafficking in glycolysis-dominant gastrointestinal tumors.

*Ganoderma polysaccharides*—fractions from *Ganoderma* species that promote DC maturation, Th1 polarization, and CD8⁺ T-cell infiltration, supporting Axis IV.

*Hochu-ekki-to*—Japanese Kampo formula used in frail, treatment-exhausted patients to support mitochondrial respiration, antioxidant defenses, and treatment tolerance.

*Huaier*—aqueous extract or granules from *Trametes robiniophila Murr.* that enhance DC maturation, IFN-γ⁺ T cells, and systemic immune competence.

*Jianpi Huayu decoction*—spleen-fortifying and liver-regulating formula that reshapes lipid–bile-acid pools, microbiota composition, and TAM/MDSC phenotypes along the gut–liver axis.

*Jianpi Jiedu decoction*—spleen-fortifying and detoxifying formula that normalizes bile-acid/lipid handling, reduces MDSCs and pro-tumor TAMs, and strengthens gut–liver barrier function.

*Kejinyan decoction*—multi-herb prescription with systems-level effects on angiogenesis, TME composition, and immune regulation.

*Quxie Capsule*—capsule formulation that reshapes intestinal and tumor metabolic milieus, decreases glycolytic signatures, and restores immune surveillance in colitis-associated colorectal cancer.

*Shenqi Yiqi Capsule (SQYC)*—Qi-tonifying capsule that enhances DC and T-cell activation, Th1 polarization, and CD8⁺ T-cell infiltration, especially in patients receiving chemoimmunotherapy.

*Xiayuxue decoction (XYXD)–like formulas*—liver–spleen–targeted prescriptions that modulate bile-acid and lipid metabolism and remodel gut–liver myeloid niches.

*Xihuang Pill*—classic formula with integrated effects on TME, angiogenesis, and tumor–immune regulation.

### Modern formulations and delivery platforms

*Astragalus- and ginsenoside-loaded nanoparticles*—nanocarriers that deliver Astragalus polysaccharides or Rg3 to tumors and lymphoid tissues, synchronizing ICD/ferroptosis with immune activation.

*Cell-membrane-coated/platelet-membrane–camouflaged nanomedicines*—biomimetic platforms that deplete glutathione, amplify radiotherapy-induced ICD, and enhance checkpoint blockade while minimizing off-target toxicity.

*Fe₃O₄-based nanohybrids*—iron oxide–containing constructs that promote ferroptosis, iron-dependent lipid peroxidation, and DC priming, thereby improving checkpoint blockade efficacy.

*Hydrogels (dextran–chitosan and related systems)*—injectable or in situ-forming matrices incorporating TCM-derived components to confine ICD/ferroptosis-inducing stimuli within tumors or post-surgical beds.

*Immunogenic nanovaccines*—nanoparticle-based vaccines that co-deliver tumor antigens and TCM-derived adjuvants to draining lymph nodes to enhance DC cross-priming and T-cell activation.

*Lonicera polysaccharide nanoparticles*—vesicle-like carriers delivering *Lonicera*-derived polysaccharides to lymphoid tissues to support DC and T-cell responses.

*Microneedle patches (including cantharidin and herb-loaded microneedles)*—minimally invasive skin-delivery platforms that elicit local ICD, enhance antigen release, and provide TCM-derived adjuvant signals.

*Schottky heterojunction photocatalyst–based systems*—photoresponsive composites incorporating TCM-related components to enhance ROS production, ICD, and cGAS–STING activation under local irradiation.

## Data Availability

Data sharing is not applicable to this article as no datasets were generated or analysed during the current study.

## Abbreviations

AhR: Aryl hydrocarbon receptor
AMPK: Adenosine 5′-monophosphate-activated protein kinase
CAR-T: Chimeric antigen receptor T cell
cGAS: Cyclic GMP–AMP synthase
COF: Covalent organic framework
CRC: Colorectal cancer
CTLA-4: Cytotoxic T-lymphocyte-associated protein 4
DAMP: Damage-associated molecular pattern
DC: Dendritic cell
FDG-PET: ^1^⁸F-fluorodeoxyglucose positron emission tomography
GI: Gastrointestinal
HCC: Hepatocellular carcinoma
HIF-1α: Hypoxia-inducible factor 1-alpha
ICD: Immunogenic cell death
ICI/ICIs: Immune checkpoint inhibitor / immune checkpoint inhibitors
IDO1: Indoleamine 2,3-dioxygenase 1
LDH: Lactate dehydrogenase
LN: Lymph node
MDSC: Myeloid-derived suppressor cell
NKT: Natural killer T cell
OXPHOS: Oxidative phosphorylation
PD-1: Programmed cell death protein 1
PD-L1: Programmed death-ligand 1
PI3K: Phosphoinositide 3-kinase
PS: Performance status
ROS: Reactive oxygen species
SQYC: Shenqi Yiqi Capsule
STAT3: Signal transducer and activator of transcription 3
STING: Stimulator of interferon genes
TAM: Tumor-associated macrophage
TCM: Traditional Chinese Medicine
TIL: Tumor-infiltrating lymphocyte
TME: Tumor microenvironment
TGF-β: Transforming growth factor-beta
XYXD: Xiayuxue decoction
GPX4: Glutathione peroxidase 4
HMGB1: High mobility group box 1
IFN-γ: Interferon-gamma
IL-12: Interleukin-12
KDM4D: Lysine-specific demethylase 4D
mTOR: Mechanistic target of rapamycin
P-gp: P-glycoprotein
UGT: UDP-glucuronosyltransferase
CYP: Cytochrome P450
GQD: Gegen Qinlian decoction
Rg3: Ginsenoside Rg3
Axis I: Glycolysis and lactate (tumor glycolytic flux and lactic acid burden)
Axis II: Mitochondrial integrity and ferroptosis / immunogenic cell death
Axis III: Lipid–bile-acid–centered myeloid niches
Axis IV: Dendritic cell / T-cell metabolic fitness
